# Function of SSA Subfamily of Hsp70 Within and Across Species Varies Widely in Complementing *Saccharomyces cerevisiae* Cell Growth and Prion Propagation

**DOI:** 10.1371/journal.pone.0006644

**Published:** 2009-08-14

**Authors:** Deepak Sharma, Céline N. Martineau, Marie-Thérèse Le Dall, Michael Reidy, Daniel C. Masison, Mehdi Kabani

**Affiliations:** 1 Laboratory of Biochemistry and Genetics, National Institutes of Diabetes, Digestive and Kidney Diseases, National Institutes of Health, Bethesda, Maryland, United States of America; 2 Laboratoire d’Enzymologie et Biochimie Structurales, Centre National de la Recherche Scientifique, Gif-sur-Yvette, France; 3 Laboratoire de Microbiologie et Génétique Moléculaire, Centre National de la Recherche Scientifique, Institut National de la Recherche Agronomique, AgroParisTech, Thiverval-Grignon, France; Fred Hutchinson Cancer Research Center, United States of America

## Abstract

**Background:**

The cytosol of most eukaryotic cells contains multiple highly conserved Hsp70 orthologs that differ mainly by their spatio-temporal expression patterns. Hsp70s play essential roles in protein folding, transport or degradation, and are major players of cellular quality control processes. However, while several reports suggest that specialized functions of Hsp70 orthologs were selected through evolution, few studies addressed systematically this issue.

**Methodology/Principal Findings:**

We compared the ability of Ssa1p-Ssa4p from *Saccharomyces cerevisiae* and Ssa5p-Ssa8p from the evolutionary distant yeast *Yarrowia lipolytica* to perform Hsp70-dependent tasks when expressed as the sole Hsp70 for *S. cerevisiae in vivo*. We show that Hsp70 isoforms (i) supported yeast viability yet with markedly different growth rates, (ii) influenced the propagation and stability of the [*PSI^+^*] and [*URE3*] prions, but iii) did not significantly affect the proteasomal degradation rate of CFTR. Additionally, we show that individual Hsp70 orthologs did not induce the formation of different prion strains, but rather influenced the aggregation properties of Sup35 *in vivo*. Finally, we show that [*URE3*] curing by the overexpression of Ydj1p is Hsp70-isoform dependent.

**Conclusion/Significance:**

Despite very high homology and overlapping functions, the different Hsp70 orthologs have evolved to possess distinct activities that are required to cope with different types of substrates or stress situations. Yeast prions provide a very sensitive model to uncover this functional specialization and to explore the intricate network of chaperone/co-chaperone/substrates interactions.

## Introduction

Seventy-kilodalton heat-shock proteins (Hsp70) are ubiquitous molecular chaperones that play essential housekeeping functions in protein folding, synthesis, assembly, transport across biological membranes and degradation. They are also involved in quality control processes, such as protein refolding after a stress injury, and control the activity of regulatory proteins in signal transduction and cell proliferation pathways [1]. All of these cellular activities depend on the ATP-regulated ability of Hsp70 s to interact with exposed hydrophobic surfaces of proteins. Hsp70 family members share a highly conserved modular structure consisting in an N-terminal 44-kDa ATPase domain (also named adenine nucleotide binding domain or NBD), an 18-kDa substrate-binding domain (SBD) and a C-terminal 10-kDa lid domain. The interaction of Hsp70 s with exposed hydrophobic stretches of protein substrates is regulated by adenine nucleotides. In the ATP-bound state, the SBD is in an open conformation resulting in low affinity for substrates. ATP hydrolysis triggers a conformational change of the lid that closes on the substrate and traps it within the SBD [2]–[4]. The exchange of ADP for ATP allows the return to an open-state and facilitates substrate release. The allosteric regulation of Hsp70 s is tightly regulated by various cochaperones. Members of the universal Hsp40/DnaJ-family stimulate the weak intrinsic ATPase activity of Hsp70 s and coordinate it with substrate binding, whereas ADP/ATP exchange is catalyzed by the unrelated GrpE, Bag1, Fes1/HspBP1 and Sse1/Hsp110 families of nucleotide exchange factors (NEFs) [1], [5], [6].

Hsp70 s constitute one of the most conserved protein families, and members of this family may be constitutively expressed or stress-inducible [7]–[12]. Hsp70 s of bacteria and humans are structurally superimposible and retain 50% amino acid identity [7], [9], [10]. Hsp70 s from different species share functional redundancy as shown by replacing endogenous Hsp70s with those from other species. For example, *Drosophila* Hsp70 efficiently protected mammalian cells from heat stress [13] and human Hsp70 was able to complement the cytoprotective function of rodent Hsp70 both *in vitro* [14]–[16] and in transgenic animals [17]–[19]. Nevertheless, such complementation is often incomplete, which probably is explained by differences in functional interactions of Hsp70s with co-chaperones and other components of chaperone machines among the different species [20]. Most organisms contain multiple members of the Hsp70 family in virtually all cellular compartments [11]. Intriguingly, in eukaryotes, while organelle specific Hsp70s are generally encoded by a single gene, multiple highly homologous Hsp70 paralogs coexist in the cytosol [11]. For example, yeast contains seven cytosolic Hsp70s, among which four SSA-subfamily members (Ssa1–4) and three ribosome-associated Hsp70s (Ssb1–2p and Ssz1p). Similarly, fruit fly and human cells contain ten and seven bona fide Hsp70s, respectively [11], [21].

Because of their high degree of conservation [7] and because only one Ssa protein is sufficient to allow yeast viability if expressed at sufficiently high levels [22], it has been long postulated that these Hsp70s were functionally redundant, only differing by their spatiotemporal expression patterns [11]. However, several recent studies (reviewed in [11]) pinpointed exquisite differences among Hsp70 orthologs suggesting that while they share redundant house-keeping functions, they may exhibit functional specificities that have yet to be fully deciphered.

Evidence for such functional specialization among Hsp70 paralogs from our laboratories emerged from studies on the propagation of the [*URE3*] and [*PSI^+^*] prions and biofilm formation in yeast. Yeast prions are self-replicating infectious aggregates of cellular proteins that cause distinct non-Mendelian phenotypes [23], [24]. The [*PSI^+^*] and [*URE3*] traits are caused by the prion properties of the Sup35p and Ure2p proteins, respectively [25]–[30]. Molecular chaperones of the Hsp40 and Hsp70 families ([31], [32] for review), as well as the Sse1/Hsp110 and Fes1/HspBP1 NEFs [33]–[35] play critical roles in prion formation and propagation. The overexpression of Ssa1p, but not Ssa2p, cures [*URE3*] [36], whereas mutations in *SSA2*, but not *SSA1*, impair [*URE3*] propagation [37]. Remarkably, yeast strains expressing individual Ssa proteins have markedly different prion phenotypes [38]. We have recently reported that the Hsp70 machinery is required for biofilm (or ‘mat’) formation in yeast [39]. Specifically, an *ssa1*Δ mutant, in an otherwise wild-type background for the other Ssa-encoding genes, is more affected in mat formation than an *ssa2*Δ mutant that has more subtle defects in this process [39]. Moreover, we showed that the additional deletion of *SSA3* or *SSA4* enhances the mat formation defects of the *ssa1*Δ and *ssa2*Δ mutants, suggesting functional cooperation between constitutive and inducible Hsp70s [39].

Here we used a previously described yeast reporter strain [38], [40] to systematically compare the ability of eight Ssa proteins from the yeasts *Saccharomyces cerevisiae* and *Yarrowia lipolytica* to allow yeast growth, prion propagation and proteasomal degradation of a model protein. Our data suggest that functional differences among cytosolic Hsp70s provide yet another way to control and fine tune their cellular activity that may be required to cope with various substrates, functions and adverse conditions.

## Results

### Nomenclature and conditional expression of Yarrowia lipolytica Hsp70 orthologs

We have recently reported that the yeast *Yarrowia lipolytica* contains four orthologs of the Ssa subfamily of Hsp70 molecular chaperones in the cytosol [11]. These proteins are highly conserved (Table 1 and Supplemental Figure S1) and it was therefore impossible to identify which of them corresponded to *S. cerevisiae* Ssa1p-Ssa4p solely based on reciprocal Blast analysis (data not shown). To avoid nomenclature confusion and for the sake of clarity, we arbitrarily chose to name the *Y. lipolytica* Ssa-type proteins Ssa5p to Ssa8p [11]. It is noteworthy that the Ssa5p-Ssa8p proteins share a higher degree of conservation with each other (92–95% identity) than the Ssa1p-Ssa4p proteins (79–97% identity) (Table 1). The *Yarrowia lipolytica* cytosol also contains orthologs of the ribosome-associated Ssb1/2p and Ssz1p proteins that we named *Yl.*Ssb1p (locus: YALI0A00132g; UniProt ID: Q6CIA7) and *Yl.*Ssz1p (locus: YALI0B12474g; UniProt ID: Q6CEW0) [11]. While a detailed molecular analysis of the *Y. lipolytica* Hsp70 machinery will be described elsewhere (Martineau C.N., LeDall M-T., Beckerich J-M. and Kabani M., unpublished data), it was essential for this study to characterize the expression patterns of Ssa5p-Ssa8p, as functional differences have been previously pointed out between constitutive and inducible Hsp70s [40]. To this aim, we documented the expression of each isoform in different growth conditions by RT-PCR analysis.

**Table 1:** Percent amino acid identity of Ssa proteins.

	Ssa1	Ssa2	Ssa3	Ssa4	Ssa5	Ssa6	Ssa7
Ssa2	97						
Ssa3	79	79					
Ssa4	81	81	87				
Ssa5	82	81	80	82			
Ssa6	81	81	80	82	94		
Ssa7	83	82	80	81	95	95	
Ssa8	81	81	81	82	93	92	92

**Figure 1:**
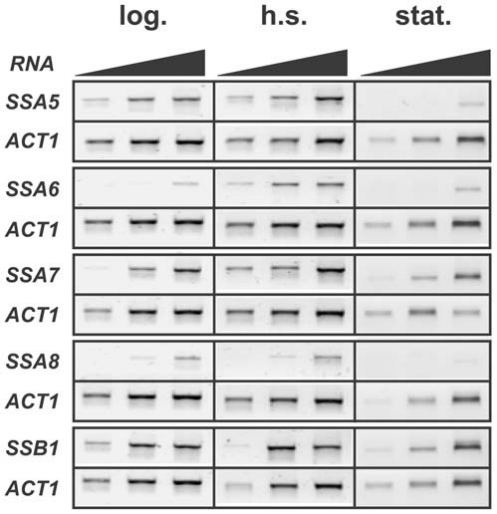
RT-PCR analysis of Hsp70 isoforms expression in *Yarrowia lipolytica*. Total RNA was isolated from a wild-type *Y. lipolytica* strain grown to early exponential phase at 28°C (left panel), followed by one hour shift at 42°C (middle panel) or left to grow to stationary phase (right panel). The expression of each Hsp70 ortholog was assessed by RT-PCR analysis (using 1, 10 or 100ng of RNA) as described in Materials and Methods using the *ACT1* gene as a loading control.

First, a wild-type *Y. lipolytica* strain was grown at 28°C and RNA was prepared from samples taken at early exponential phases. RT-PCR was performed to detect each Hsp70 isoform or the actin-encoding gene *ACT1* as a control. As shown in Figure 1, *SSA5*, *SSA7* and *Yl.SSB1* were expressed in exponentially growing cells, whereas *SSA6* and *SSA8* were only modestly detected. As the cells progress in stationary phase, we barely detected *SSA5* and *SSA8* mRNAs, *SSA6* levels remained very low, whereas *SSA7* and *Yl.SSB1* mRNA levels were still easily detectable at the lowest RNA concentration used for the assay (Figure 1). It should be noted that the *ACT1* mRNA levels substantially decreased in stationary phase compared to exponential phase, which is consistent with earlier observations in *S. cerevisiae* or *Candida albicans* [41]–[43].

To assess stress inducibility, the same 28°C cultures were heat-shocked for one hour at 42°C, before RNA was extracted. In these conditions, *SSA6* and *SSA7* were up-regulated ∼4–5 fold and ∼1,5–2 fold, respectively, whereas the levels of *SSA5*, *SSA8*, and *Yl.SSB1* were not significantly affected (Figure 1 and data not shown). Similar results were obtained when the cells were shifted to 37°C, a temperature at which *Y. lipolytica* cells are unable to grow but can nevertheless survive for several hours (data not shown). Thus, from these data, we expect Ssa6/7p to be functionally similar to the inducible Ssa3/4p, whereas Ssa5/8p would be more similar to the constitutive Ssa1/2p. Finally, we can not exclude that the expression of the *Y. lipolytica* Hsp70 encoding genes is also modulated by yet unidentified environmental cues and stress injuries.

### Growth of S. cerevisiae cells expressing individual Ssa proteins varies widely

The *SSA* subfamily of Hsp70 molecular chaperones is essential for growth of *S. cerevisiae* [22]. Our earlier work with strains lacking all four chromosomal *SSA* genes and expressing individual Ssa proteins from plasmids under control of the *SSA2* promoter confirmed that Ssa1p, Ssa2p, Ssa3p and Ssa4p each support cell growth [22], [38]. To extend this study we systematically compared the ability of individual Hsp70s from the yeasts *S. cerevisiae* (Ssa1p-Ssa4p) and *Y. lipolytica* (Ssa5p-Ssa8p) to handle known Hsp70-dependent tasks *in vivo*. We expected functional differences among Hsp70 isoforms to be subtle and mainly depend on different substrate preferences and/or different affinities for cochaperones. Because of their evolutionary distance, we expected that the *Y. lipolytica* Hsp70 proteins may interact less efficiently with *S. cerevisiae* cochaperones and that any functional differences between isoforms will therefore be exacerbated in this heterologous context.

**Figure 2:**
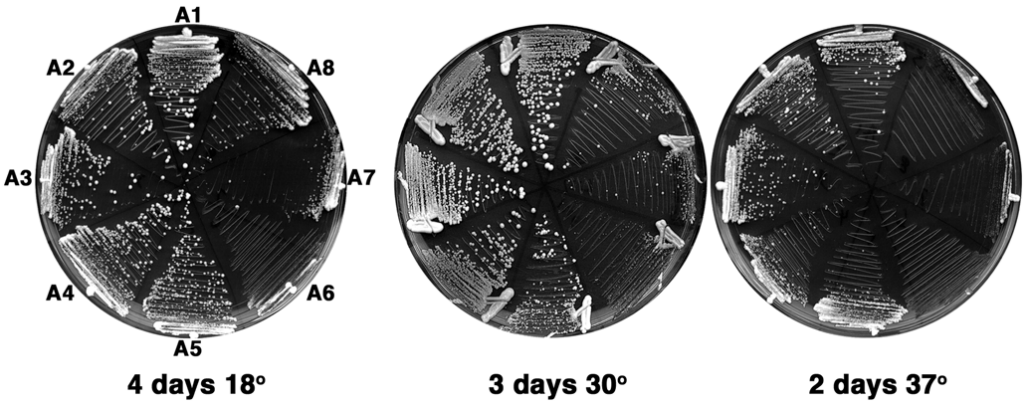
Growth of *S. cerevisiae* expressing individual Ssa proteins. G402 strains A1–A8 [*psi^−^*] cells were streaked onto YPAD plates and incubated at the indicated temperature for 2–4 days as indicated. Strains are as indicated on first panel, pattern of strains is the same on each plate.

The *LEU2*-based plasmids pA1, pA2, pA3 and pA4 that allow the expression of *SSA1, SSA2, SSA3,* and *SSA4* under the control of the strong and constitutive *SSA2* promoter, respectively, were described previously [38], [40]. The pA5, pA6, pA7 and pA8 vectors that allow the expression of *SSA5, SSA6, SSA7* and *SSA8* under the control of the *SSA2* promoter, respectively, were similarly constructed (see Materials and Methods). These plasmids were used to transform strain G402, which lacks all chromosomal *SSA* genes and expresses *SSA1* from the *URA3-*based plasmid pRDW10 [44]. The ability of the pA1-A8 plasmids to support growth of G402 was assessed by challenging transformants on medium containing 5-fluoro-orotic acid (5-FOA) to counter-select against plasmid pRDW10. The resulting Ura^−^, Leu^+^ strains expressing each individual Ssa protein were hereafter referred to as A1 to A8. All *SSA* genes supported yeast growth, albeit with markedly different growth rates indicating significant functional differences among these Hsp70 orthologs (Table 2 and Figure 2). As expected, *Yl.*Ssb1p was not able to support yeast growth, nor was an Ssa4p mutant with a D393G mutation in the conserved linker that is predicted to impair the inter-domain communication between the NBD and the SBD (Table 2). In rich liquid medium at optimal temperature A1 and A2 grew at near wild type rate, A3 cells grew somewhat more slowly and A4 cells took roughly 30% longer to divide (see Table 2). All of the *Y. lipolytica* Hsp70s supported cell growth but none as well as the *S. cerevisiae* Ssa proteins and there was more variability in growth rates. For example, A5 cells grew best, yet slightly more slowly than those expressing Ssa4p, and A6 cells took three times longer than wild type cells to double in numbers. Importantly, strains expressing inducible Hsp70s (A3, A4, A6 and A7) grew more slowly than their constitutive counterparts (A1, A2, A5 and A8), respectively. These results agree with our previously published data showing inability of primate inducible Hsp70 to confer yeast viability [40], and indicate that stress-inducible Hsp70s may be partly or totally unable to perform important house-keeping functions that require more efficient activities possessed by the constitutive isoforms.

**Table 2:** Growth rates and prion phenotypes of strains expressing individual Ssa proteins.

		Prion phenotype^b^	
Hsp70 isoform	Growth rate^a^ (min/cell div)	[*PSI* ^+^]	[*URE3*]
Ssa1p	110	++++	++
Ssa2p	107	+++	++++
Ssa3p	117	+++++	+/−
Ssa4p	141	++	+
Ssa5p	153	++++	+++
Ssa6p	306	+/−	−
Ssa7p	179	+/−	+
Ssa8p	164	+++	+++
*Yl.*Ssb1	inviable	na	na
Ssa4^D393G^	inviable	na	na

a Cells were grown in 15 ml of YPAD in 50 ml flasks shaking at 220 rpm at 30°. Our isogenic wild type strains 779-6A and 1075 have cell division time of 100 minutes.

b Based on data from Figures 5 and 9 and shown relative to those of typical wild type cells (++++, exemplified by cells expressing Ssa1p for [*PSI^+^*] and Ssa2p for [*URE3*]); na, not applicable (Hsp70 did not support growth).

Differences in growth were more pronounced when strains were grown on solid medium at different temperatures (see Figure 2). All strains expressing *Y. lipolytica* Hsp70s were temperature sensitive at 37°C, which might reflect reduced ability of these Hsp70s to protect important cellular factors or possibly a lower thermal stability of Hsp70s that evolved with this organisms’s lower optimal and maximal growth temperatures. Strain A6 was unable to grow while A7 and A8 were viable but did not form noticeable colonies after 2 days. Growth of A6 was also most sensitive to reduced temperature. Thus, although sequence identity of Ssa5p-Ssa8p is very similar to Ssa1p-Ssa4p, they functioned differently than all four *S. cerevisiae* Hsp70s in supporting growth. Additionally, although Ssa5p–Ssa8p are very highly homologous to each other, the considerable differences in their abilities to support growth of *S. cerevisiae* demonstrates clear functional distinctions among them.

### Inducible Hsp70s provide increased thermoresistance

A major role played by Hsp70 molecular chaperones is to protect cells from stress injuries by preventing aggregation of partially unfolded proteins. Additionally, Hsp70 and Hsp40 cooperate with Hsp104, a molecular chaperone found in microorganisms and plants that acts by resolubilizing aggregated proteins [20], [45] and is also required for [*PSI^+^*] propagation [46]. The survival rate of yeast cells to a lethal heat-shock can be greatly improved by pre-treating the cells with a mild non-lethal heat-shock, which stimulates the heat-shock and stress response pathways leading to elevated expression of heat-shock proteins (including Hsp70s and Hsp104) [12], [47].

**Figure 3:**
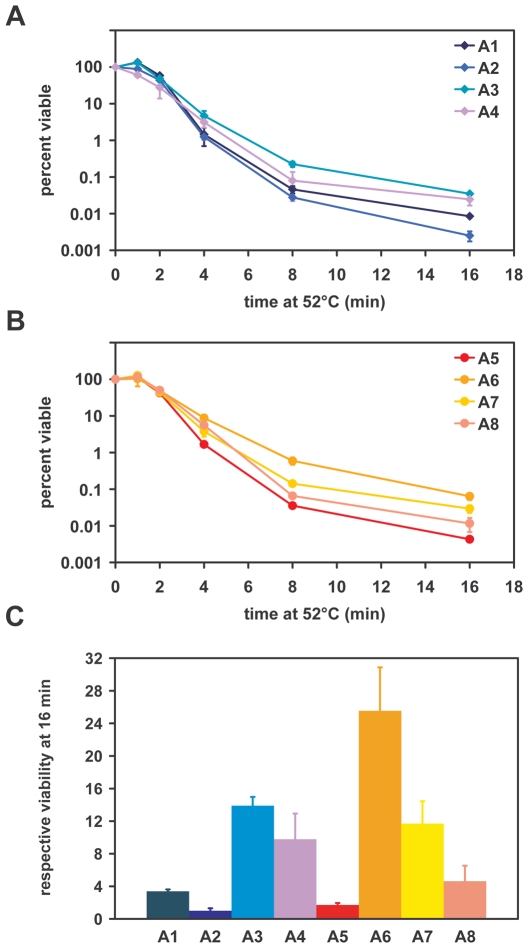
Thermoresistance properties of strains expressing individual Ssaps. The indicated strains were grown to early exponential phase at 28°C then shifted at 52°C. (A, B) Aliquots were periodically removed and cell viability was assessed by plating tenfold serial dilutions onto YPAD plates. (C) The viability of each strain at the 16 min time point was expressed as a fold enrichment over that of A2 which was arbitrarily set to 1.

We compared the thermoresistance of the A1–A8 strains exposed to a lethal heat-shock at 52°C [40] in the absence of a mild heat-shock pre-treatment to avoid any differences between Hsp70 isoforms being masked by the up-regulation of other chaperones. As shown in Figure 3, the thermoresistance of all strains was comparable, except that the strains expressing inducible Hsp70 isoforms (A3, A4, A6, and A7) were significantly more resistant than those expressing constitutive Hsp70s. After 16 min at 52°C, ∼10 to 14-fold more heat-resistant cells were recovered for the A3, A4 and A7 strains than for A2, which was the least resistant strain; the A6 strain showed the greatest survival rate with a 25-fold increase in the number of surviving cells compared to A2 (Figure 3C). This result indicates that inducible Hsp70s may be more efficient in cooperating with Hsp104 during the process of protein disaggregation and/or have more affinity with heat-denatured substrates than their constitutive counterparts. It is noteworthy that the A6 and A7 strains, which grow very slowly, were nevertheless as thermoresistant as the A3 and A4 strains (Figure 3C). This result indicates that the role of Ssa6p and Ssa7p in protecting cells from stress is fully conserved in this heterologous context and that the reason why it poorly supports cell viability is probably due to its inability to replace constitutive Hsp70s in one or more house-keeping pathways.

### Individual Ssaps vary in their ability to help proteins refold in vivo

**Figure 4:**
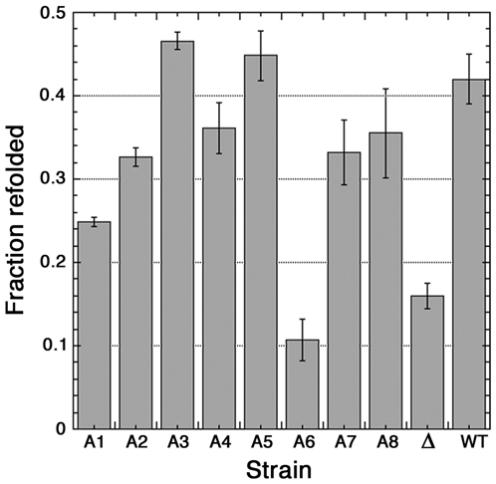
Influence of Ssa proteins on refolding of luciferase. Cultures of strains expressing a thermolabile luciferase and individual Ssa proteins (indicated at bottom) were shifted from 30°C to 37°C for 30 minutes and then to 42°C for one hour. Luciferase activity, expressed as a fraction of pre-heat shock activity, was measured after allowing cells to recover for 30 minutes at 25°C. Cychloheximide was added 50 minutes after shifting to 42°C to prevent synthesis of luciferase during the recovery period. Wild-type and *hsp104*Δ (Δ) strains (with intact *SSA1*–*4* genotype) were used as controls. Data are averages ± standard deviation from at least two cultures for each strain measured in triplicate.

The ability of chaperones to help proteins refold in vivo can be assessed by monitoring reactivation of a thermolabile bacterial luciferase after exposing cells to elevated temperature. When elevated temperature causes widespread protein aggregation, Hsp104 becomes critical for survival by disaggregating proteins in a reaction dependent upon Hsp70 and Hsp40 [20]. We monitored luciferase reactivation in strains A1-A8 by shifting exponentially growing cells from 30°C to 37°C for 30 minutes to induce expression of heat shock proteins, including Hsp104, and then to 42°C to cause extensive aggregation of luciferase. After this treatment the cells were allowed to recover at 25°C for 30 minutes. We measured luciferase after this recovery period and compared it with the activity in cells before the heat treatment (see Material and Methods). In wild type cells, over 40% of luciferase activity was restored, while in cells lacking Hsp104 only 15% was reactivated (Figure 4). Cells expressing Ssa6p were like cells lacking Hsp104, which would explain why A6 cells do not stably propagate prions (see below). Cells expressing Ssa1p had reduced reactivation function. Luciferase reactivation in the A2, A4, A7 and A8 strains was at or near wild type levels, while A3 and A5 consistently performed better than all the others (Figure 4).

### [PSI^+^] propagation is highly variable in cells expressing individual Hsp70 orthologs

Strains G402 and 1161 allow us to monitor the prions [*PSI^+^*] (this section) and [*URE3*] (see below), respectively, by a white/red color phenotype and a requirement of adenine for growth. Cells with prions are white, those without prions are red (see Materials and Methods). The strength of prion phenotype is proportional to the extent that Sup35p or Ure2p is depleted into insoluble prion aggregates [48], which is reflected in the extent of pigment accumulation and growth without adenine. Weaker prion propagation is seen as intermediate (pink) colony color and/or slower growth without adenine. The stability of prions in a growing population requires continuous replication of prions in order to keep pace with cell divisions. Reduced prion replication can be seen as spontaneous appearance of red colonies in a population of cells spread on solid medium.

**Figure 5:**
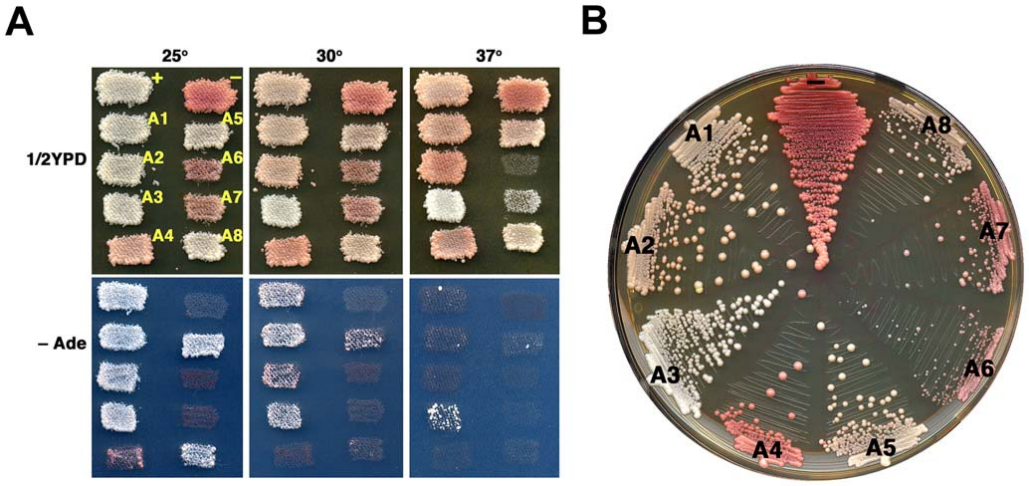
[*PSI^+^*] prion phenotype of cells expressing different Ssa proteins. (A) Patches of G402 strains A1-A8, as indicated, were grown over night at 30° on 1/2YPD and then replica-plated onto 1/2YPD plates and SD plates without adenine (–Ade), which were then incubated at the indicated temperatures. Plates at 25° were incubated three days. Plates at 30° were incubated two days. At 37° the 1/2YPD plate was incubated two days and the –Ade plate for three days. The whiter appearance of cells at 37° for strains A7 and A8 is due to reduced growth, which has not depleted enough adenine from the medium to cause pigment accumulation. (B) Streaks of cells from the same cultures used in (A) were incubated two days at 30° followed by three days at 25°. Red colonies in streaks arise from cells that lost [*PSI^+^*] before forming the colony. Red sectors in pink or white colonies are progeny of cells that lost [*PSI^+^*] during growth of the colony. In both (A) and (B) all strains are [*PSI^+^*] except A6 and A7, in which [*PSI^+^*] is very unstable (see text). [*PSI^+^*] (+) and [*psi^−^*] (−) variants of wild type strain 779-6A are included for comparison.

Earlier we showed that [*PSI^+^*] strength varied in cells expressing Ssa1p-Ssa4p proteins individually [38]. Here we confirm these results (see Figure 5) showing [*PSI^+^*] was normal in cells expressing Ssa1p, as seen by white color on 1/2YPD and good growth on –Ade at 30°C. [*PSI^+^*] was slightly weaker in cells expressing Ssa2p, as seen by pinker color on all media and weaker growth on –Ade, especially with increasing temperature, which weakens prions independently of other factors [38]. [*PSI^+^*] was stronger than normal in cells expressing Ssa3p, which remained ade+ and white even at 37°C, and weakest in cells expressing Ssa4p, which were pink and slow growing on –Ade even at 25°C. We find that strength of [*PSI^+^*] phenotype in strains A5-A8 also varied (see Figure 5). [*PSI^+^*] phenotype of strain A5 was similar to that of A2, and [*PSI^+^*] in strain A8 was somewhat weaker still but stronger than in strain A4. Although we were able to isolate [*PSI^+^*] variants of strains A6 and A7, the prion was very weak and unstable. Combined with slower growth, especially of strain A6, it was difficult to characterize [*PSI^+^*] phenotype in these strains.

Mitotic transmission of [*PSI^+^*] was uniformly stable (see Figure 5B) in most of the strains with minor exceptions. Although freshly subcultured A5 and A8 strains displayed normal [*PSI^+^*] stability, when they were retrieved from 15% glycerol stocks stored at −80°C, [*psi^−^*] cells appeared at frequencies of 5–20%. Currently we have no explanation for this instability. It is possible the weakened state of the prion is more susceptible to changes in chaperone levels during freezing and thawing. If so, then the mechanisms causing the weakening of [*PSI^+^*] under normal growth conditions in these strains would be qualitatively different than the weakening in A2 or A4 cells, in which [*PSI^+^*] is stable from storage.

### Individual Ssa proteins do not induce formation of different [PSI^+^] prion variants

A remarkable property of prion-prone proteins is their ability to generate structurally distinct prion strains, or variants, from the same polypeptide sequence that lead to distinct heritable phenotypes [32], [49]. Purified Sup35p spontaneously assembles into self-seeding, β-sheet-rich amyloid fibers *in vitro* [50], [51]. When introduced in [*psi^−^*] cells, these fibers efficiently convert them to the [*PSI^+^*] state [52]–[54]. Sup35p fibers adopt various conformations *in vitro* that trigger the formation of phenotypically distinct [*PSI^+^*] cells upon transformation into [*psi^−^*] cells [53]–[55]. Similarly, when cell extracts prepared from weak or strong [*PSI^+^*] variants are introduced into [*psi^−^*] cells, the resulting [*PSI^+^*] cells retain the original prion characteristics [53], [54]. Earlier work showed that altered chaperones can change the way the same prion variant propagates [48], [56] or cause prions to evolve into variants with different propagation properties [57]. The weak [*PSI^+^*] phenotypes of some strains (e.g. A4) could be caused by weakened propagation of the same prion variant or by the formation of a different prion variant

**Figure 6:**
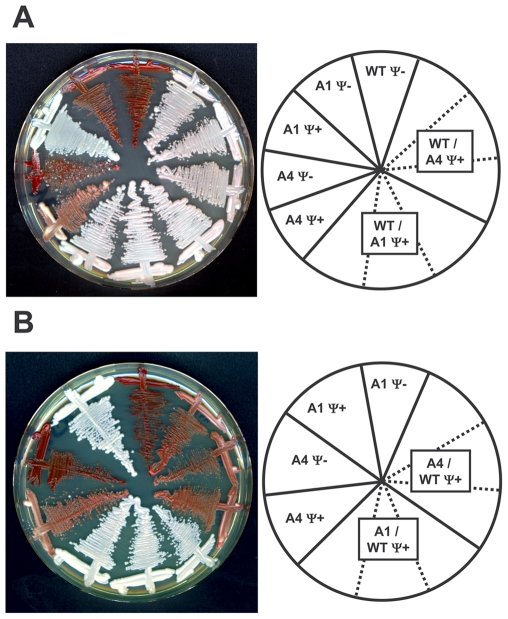
Strains expressing individual Ssaps do not generate different prion strains. (A) The wild-type 74D-694 [*psi^−^*][*pin^−^*] strain was transformed with crude extracts from the A1 [*PSI^+^*] and A4 [*PSI^+^*] strains as described in Materials and Methods. Induced 74D-694 [*PSI^+^*] colonies were isolated and their prion phenotype assessed on 1/2 YPD plates along with the [*psi^−^*] and [*PSI^+^*] derivatives of the A1 and A4 strains. For each transformation, three independent [*PSI^+^*] transformants are shown (their positions are indicated by dashed lines on the right panel) (B) The A1 [*psi^−^*] and A4 [*psi^−^*] strains were transformed with a crude cell extract from the 74D-694 [*PSI^+^*][*pin^−^*] strain. The prion phenotypes of representative [*PSI^+^*] transformants were assessed on 1/2 YPD plates and compared to those of the original A1 and A4 strains. For each transformation, three independent [*PSI^+^*] transformants are shown (their positions are indicated by dashed lines on the right panel). In each case, the plates were incubated for 3 days at 28°C and 4 days at 23°C.

To test the possibility that different, structurally distinct, prion variants are generated in the presence of different Hsp70 orthologs, we focused on A1 and A4 that have normal and weak prion phenotypes, respectively (Figure 5A). We prepared crude cell extracts from the A1 [*PSI^+^*] and A4 [*PSI^+^*] strains and used them, together with the *URA3-*based pRS316 plasmid, to transfect spheroplasts derived from the [*psi^−^*][*pin^−^*] 74D-694 strain (see Material and Methods section; [58]). [*PSI^+^*] cells were then selected among Ura+ transformants (see Material and Methods). If the strong and weak [*PSI^+^*] of the A1 and A4 strains correspond to structurally different prions, then we expect these prion characteristics to be transmitted to a wild-type strain [53], [54]. In turn, if A1 and A4 have structurally similar prions, then we expect the prion phenotypes of a wild-type strain induced by the corresponding cell extracts to be similar [53], [54]. As shown in Figure 6A, the prion phenotypes of 74D-694 [*PSI^+^*] cells obtained after transformation with the A1 [*PSI^+^*] or the A4 [*PSI^+^*] extracts were very similar, indicating that A1 and A4 do not contain structurally different prion strains. It should be noted that [*PSI^+^*] is slightly stronger in A1 than in the converted 74D-694 [*PSI^+^*] strains (Figure 6A). As expected, no [*PSI^+^*] colonies were obtained when 74D-694 was transfected with crude cell extracts prepared from the A1 [*psi^−^*] or the A4 [*psi^−^*] strains (data not shown).

The [*PSI^+^*] variant in all our strains originated in the parental G402 strain prior to the replacement of pRDW10 by pA1 or pA4. Since the different Ssa proteins did not induce formation of new prion variants, the 74D-694 transfectants should carry this original prion variant and we should be able to reproduce the prion phenotypes of A1 and A4 using seeds from these 74D-694 [*PSI^+^*] cells. To confirm this conclusion, we used crude cell extracts from a strong [*PSI^+^*][*pin^−^*] derivative of strain 74D-694 to transfect [*psi^−^*] derivatives of A1 and A4 as described above. As shown in Figure 6B, the prion phenotypes of the transfected strains were very similar to those observed for the original [*PSI^+^*] strains: the A1 and A4 strains retained their strong and weak prion phenotypes, respectively. These data show that the prions in A1 and A4 are the same and the differences in [*PSI+*] phenotype are due to different effects of Hsp70 orthologs on the way this prion propagates.

### Ssa Hsp70s variously affect size of prion polymers

**Figure 7:**
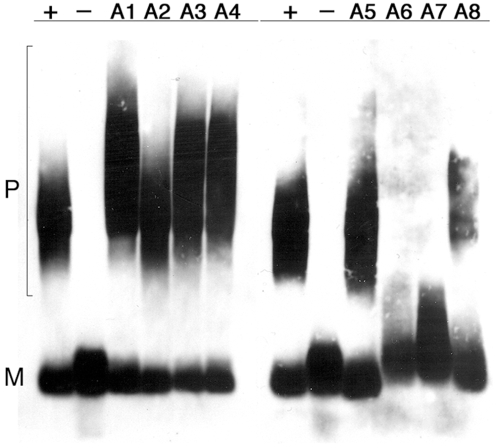
SDD-AGE analysis of Sup35p polymers in G402 [*PSI^+^*] cells. After inducing expression of Sup35-GFP fusion protein (NGMC) for 15 hours, log phase cells were harvested and cell lysates were incubated in SDS to dissolve all but the prion polymers. The lysates were then separated on agarose gels and immunoblotted using anti-GFP antibodies. Strain names are indicated at top. Horizontal line at top indicates origin. A1-A4 were processed separately from A5-A8. Wild type 779-6A [*PSI^+^*] and [*psi^−^*] controls processed independently for each blot show consistency between the blots. The high molecular weight smear represents polymers (P) of NGMC, faster migrating NGMC monomer (M) is at the lower part of the blot.

The ways the different Ssa proteins affected prion strength and stability could be due to effects on prion growth or replication (polymer fragmentation). For example, Ssa2p and Ssa4p reduced [*PSI^+^*] strength but did not affect stability, suggesting they reduce incorporation of Sup35p into [*PSI^+^*] polymers but did not affect prion replication. By monitoring migration of prion polymers on semi-denaturing agarose gels (SDD-AGE), earlier work showed that inhibiting processes required for polymer fragmentation caused the average length of polymers to increase [59]. The size of polymers could also increase, however, if addition of monomers to polymers is enhanced while the rate of fragmentation remains unchanged. Thus, increased polymer size reflects either reduced fragmentation or enhanced growth. We used SDD-AGE to determine if the size of prion polymers in strains expressing the various Ssa proteins would provide an indication of whether prion growth or replication was being affected (Figure 7). We grew cells in rich medium at 30°C and used a previously described plasmid-borne Sup35-GFP fusion protein (NGMC, [60]) so we could simultaneously monitor prion aggregation status in live cells (see below). Although we do not know whether this fusion protein incorporates within polymers of the endogenous Sup35p, the fusion protein is induced to form prions by the endogenous [*PSI^+^*] and retains the propagation characteristics of the prions that induce it [60].

All the Sup35-GFP from our wild type [*psi^−^*] strain with the intact *SSA* genotype migrated as monomer while the [*PSI^+^*] control showed less monomeric Sup35-GFP and a significant amount of Sup35-GFP migrating as a smear of high molecular weight material (Figure 7). Surprisingly, the strains with patterns most like wild type were A2, A5, and A8, all of which had weaker phenotypes. The apparent relative amount of monomer, however, correlated with phenotype. For example, A8 had the weakest phenotype and, by visual estimate, the highest relative amount of monomer. One possible explanation for these data is that A2, A5 and A8 modestly inhibit both fragmentation and growth of polymers to levels that produce fewer polymers but with a typical size range. Although A3 and A4 had opposite effects on the [*PSI^+^*], both had polymers that were larger than normal (Figure 7). For A3, the data could be explained if replication remained efficient but Sup35-GFP was incorporated into prion polymers at a faster rate, which would confer a stronger phenotype. For strain A4, it could be that growth is normal but replication is inhibited, which would lead to larger but fewer polymers to deplete Sup35-GFP, and thus confer the weak prion phenotype. If so, then replication must not be reduced enough to prevent efficient transmission of prions during mitosis since [*PSI^+^*] is stable in strain A4. Overall these data suggest the different Hsp70s can affect both processes of growth and fragmentation and do so in distinct ways.

### Ssa Hsp70s variously affect aggregation of Sup35p in [PSI^+^] cells

**Figure 8:**
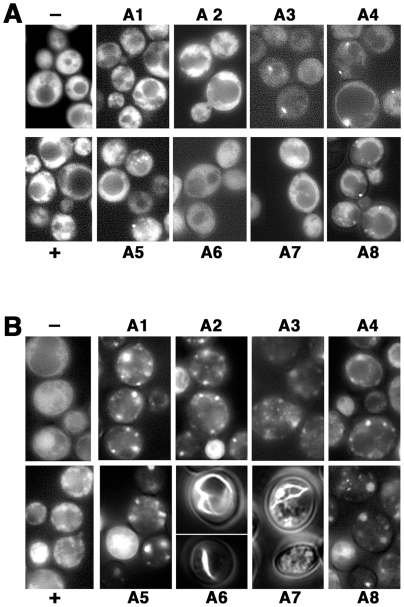
Aggregation of Sup35-GFP in vivo. (A) Fluorescent images of cells from cultures used for the SDD-AGE experiments were taken at the same time cells were processed for SDD-AGE. (B) Images of cells from the same cultures after incubation for an additional six days at 25° in the same medium. Strains A1-A8 are indicated, + and – are wild type 779-6A [*PSI^+^*] and [*psi^−^*] controls.

Although individual Sup35p polymers remain intact in the presence of SDS, any interaction between the polymers giving rise to higher-order aggregates is disrupted. To assess the overall aggregation status of Sup35p, and possibly determine the extent these polymers self associate into larger aggregates, we monitored fluorescence of Sup35-GFP in the same cells used for the SDD-AGE experiments. The fusion protein propagates like a typical [*PSI^+^*] prion and reports on the state of endogenous Sup35p [60]. Fluorescence in our wild type [*psi^−^*] cells was smoothly diffuse in the cytosol (Figure 8A). When [*PSI^+^*] was present in the same cells, fluorescence was somewhat granular in appearance, indicative of Sup35-GFP aggregation.

Aggregation of Sup35-GFP in cells expressing individual Ssa proteins was more intense and varied depending on the isoform being expressed (Figure 8A). While fluorescence in A1 and A2 cells resembled that of wild type cells, A3 and A4 cells had dimmer overall fluorescence and most cells contained one or two small bright foci. A5 cells had a combination of these patterns, with high overall brightness and granular appearance but also with bright foci. Fluorescence in A8 cells was similar to that of cells expressing Ssa3p or Ssa4p but more cells had multiple foci. A6 and A7 cells had mostly bright diffuse fluorescence with occasional (∼5%) cells having a granular appearance.

We and others [61] reported that cells in aging cultures show changes in prion aggregation state. We therefore observed cells in these same Sup35-GFP cultures after allowing them to remain at 25 degrees for an additional 5–6 days to see if the Ssa proteins affected these changes differently (see figure 8B). There was no significant change in fluorescence of [*psi^−^*] cells but Sup35-GFP in [*PSI^+^*] was more extensively aggregated showing a large number of more defined foci. Aggregation of Sup35-GFP was similarly increased in A1, A2 and A5 strains and these cells had many fewer but much larger aggregates. These and all the other cells expressing individual Ssa proteins had noticeably dimmer overall fluorescence. Sup35-GFP in A3 and A4 cells was not very different from log phase cells with the exception that more foci were visible. A8 cells also had larger, but dimmer foci. Thus, Sup35p in aged cells expressing any of these individual Ssa proteins aggregated similarly into a smaller number of larger foci than we saw in wild type cells.

Although we had difficulty distinguishing whether colonies of A6 and A7 cells were [*PSI^+^*] because of their very weak and unstable phenotype, aged cultures of these strains contained rare (0.5 - 2%) cells that were extremely bright and easy to identify among hundreds of dimmer cells. The fluorescence in these cells appeared as threads in web-like, branched and ring-shaped patterns. We presume that cells with these aggregates represent [*PSI^+^*] cells in the originating culture, and that some effect of a particular Ssa6p and Ssa7p activity that leads to prion instability might eventually cause this type of aggregation as cells age. Additionally the brightness of these cells suggests that Sup35-GFP accumulates to a very high level, which likely contributes to the atypical aggregation.

These data show that different Hsp70s can affect aggregation of prion proteins differently. On the whole, however, the fluorescence patterns did not correlate with the prion phenotypes, indicating that the particular aggregation state of prion protein does not reflect how the strength or stability of the prion is being affected.

### Individual Hsp70s affect [URE3] differently than [PSI^+^]

**Figure 9:**
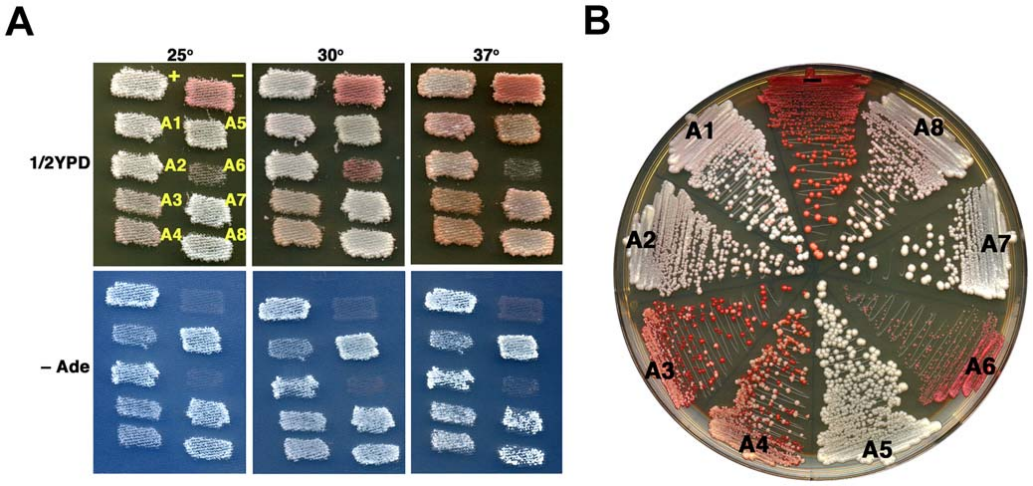
[*URE3*] prion phenotype of cells expressing different Ssa proteins. (A) Patches of 1161 strains A1-A8, as indicated (see Figure 5A), were grown on 1/2YPD and replica-plated onto 1/2YPD and –Ade plates as in Figure 5A. Plates were incubated at the indicated temperature for two days. Cells expressing Ssa6p are unable to propagate [*URE3*] and are uniformly [*ure-o*]. (B) Streaks of cells from the same cultures used in (A) were incubated two days at 30° followed by three days at 25°. Red colonies in streaks arise from cells that lost [*URE3*] before forming the colony. Red sectors in pink or white colonies are progeny of cells that lost [*URE3*] during growth of the colony. [*URE3*] (+) and [*ure-o*] (–) variants of wild type strain 1075 are included for comparison.

Earlier we showed that Ssa1p-Ssa4p affected [*URE3*] differently than [*PSI^+^*] [38]. In contrast to [*PSI^+^*], [*URE3*] is unstable in cells expressing only Ssa1p and normal in cells expressing only Ssa2p, and it is weak and very unstable in cells expressing only Ssa3p. In cells expressing only Ssa4p [*URE3*] is weak, like [*PSI^+^*], but somewhat unstable. We confirm these results (Figure 9A,B) and find that [*URE3*] phenotypes of 1161 strains A5-A8 were different than those in cells expressing only Ssa1p-Ssa4p (see Figure 9). Similar to that of A2 cells, [*URE3*] was both strong and stable in A5, A7 or A8 cells. We were unable to isolate A6 cells capable of propagating [*URE3*]. Since Ssa6p supports growth weakly and [*URE3*] reduces growth of our strains, the combination of effects might make cells inviable.

As we saw with [*PSI^+^*], [*URE3*] was unstable in the strains expressing individual Ssa proteins that were retrieved from frozen storage. Roughly 1% of A2 cells from frozen stocks were [*ure-o*], while about 5–10% of cells from A5, A7 and A8 stocks had no prion. Again, we have no explanation for this instability, which we do not observe for wild type cells with an intact *SSA* genotype (strain 1075). Thus, although prions propagated efficiently in cells expressing individual Ssa proteins, freezing and thawing revealed a requirement for specific Hsp70 function to maintain prion stability under certain conditions.

### Efficiency of [URE3] curing by Ydj1p depended on individual Ssa proteins

The very high identity of yeast Ssa proteins led us to suggest that previously observed differences in their effects on prions might be caused by differences in the way they are regulated by co-chaperones rather than by differences in their intrinsic Hsp70 activities [38]. Overexpressing Ydj1p or Ssa1p “cures” cells of [*URE3*] but increasing Ssa2p does not [36], [62]. Our earlier work showed this curing depends on interaction of Ydj1p with Hsp70 but does not require Ydj1p substrate binding activity [63], suggesting Ydj1p eliminates [*URE3*] indirectly by affecting Hsp70 function.

**Figure 10:**
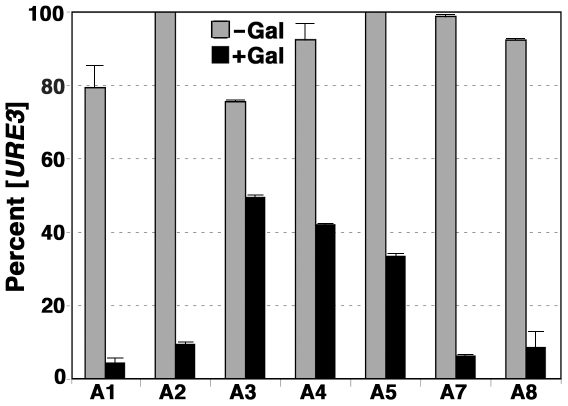
Efficiency of [*URE3*] curing by elevated Ydj1p depends on Hsp70 isoform. Cultures of 1161 [*URE3*] strains A1–A8 (except A6, which cannot propagate [*URE3*]), as indicated, were split into non-inducing (-Gal) and Ydj1p inducing (+Gal) conditions and grown to OD_600nm_  = 1–2. Aliquots were diluted to obtain 300–500 cells per plate onto 1/2YPD and the percentage of [*URE3*] cells remaining, scored as white colonies on the 1/2YPD plates, is shown. Data are averages of at least two experiments, error bars indicate standard deviation. For strains A2 and A5 we saw no loss of the prion among a total of about 1500 colonies. Overexpressing Ydj1p had no noticeable effects on fitness of any of the strains.

To test if different Hsp70s affected Ydj1p curing of [*URE3*] differently, we monitored loss of [*URE3*] after inducing expression of Ydj1p from a galactose-inducible promoter in 1161 strains A1–A8 (see Figure 10). Since the strains grew at different rates, [*URE3*] loss was scored when cells divided 6–7 times. Like wild type cells, those expressing only Ssa2p were cured of [*URE3*] very efficiently so less than 10% of cells retained [*URE3*]. Because [*URE3*] is unstable in A1 cells, only 75–80% of A1 cells had [*URE3*] even after growth in non-inducing medium. Induction of Ydj1p also cured [*URE3*] very efficiently in these cells, with less than 5% of these cells retaining [*URE3*]. In contrast, although [*URE3*] was both weak and unstable in the strain expressing only Ssa3p, overproducing Ydj1p cured [*URE3*] much less efficiently in A3 cells. Untreated A3 cultures had a similar proportion of [*URE*] cells as A1 cultures, but 65% of these still had the prion after Ydj1p overexpression. [*URE3*] also was much less sensitive to elevated Ydj1p in cells expressing only Ssa4p or Ssa5p, but was eliminated efficiently from cells expressing Ssa7p or Ssa8p (figure 10). Thus, efficiency of curing did not depend on the strength or stability of [*URE3*] before elevating Ydj1p, but depended critically on the particular Hsp70 being expressed. These data confirm that Ydj1p eliminates [*URE3*] by a mechanism that requires Hsp70 and suggest that Ydj1p interacts differently with Ssa3p, Ssa4p, and Ssa5p than with the others. The data further suggest that the weak prion phenotype in cells expressing only Ssa3p or Ssa4p is not due to a Ydj1p effect.

### CFTR degradation occurs at comparable rates in strains expressing individual Ssaps

The viability of the strains expressing individual Ssa proteins indicates that essential Hsp70-dependent functions such as protein folding, clathrin uncoating or protein translocation across the endoplasmic reticulum (ER) membrane are preserved, although with variable efficiencies as suggested by the differences in growth rates (Table 2). We then asked whether individual Ssaps would affect a specific Hsp70-dependent quality control mechanism other than prion propagation, and compared the degradation rates of the human cystic fibrosis transmembrane conductance regulator (CFTR) in our A1–A8 strains. CFTR is a polytopic plasma membrane chloride channel in which mutations cause cystic fibrosis [64]. CFTR folding at the endoplasmic reticulum (ER) membrane is a very inefficient process and most (∼70–80%) of the wild-type protein is degraded by the proteasome through ER-associated degradation (ERAD), while a minor (20–30%) fraction of the protein pursues its route to the plasma membrane [65], [66]. When expressed in yeast, wild-type CFTR is entirely retained at the ER membrane and degraded through ERAD in a process that requires Hsp70 function [67].

**Figure 11:**
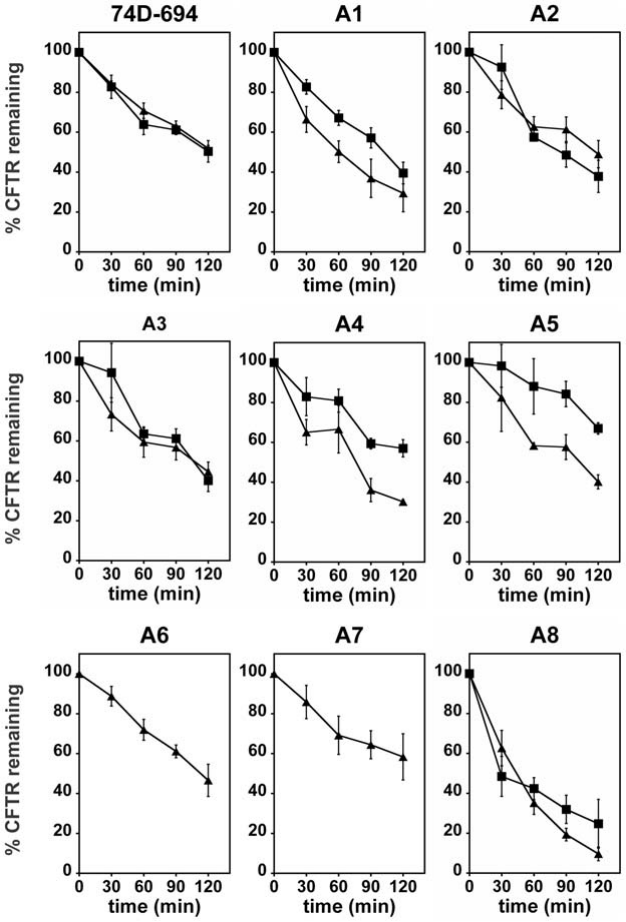
CFTR degradation monitored by cycloheximide-chase. The indicated strains, either [*psi^−^*] (triangles) or [*PSI^+^*] (squares), were grown to early log phase at 28°C, then cycloheximide was added at a final concentration of 200 µg/mL. Aliquots were taken periodically and total protein extracts were prepared and subjected to SDS-PAGE and immunoblot analysis as described in Materials and Methods. Immunoblots were quantified by PhosphorImager analysis and the amount of CFTR at time zero was set to 100% (representative blots for each strain are shown on Supplemental Figure S2). Error bars represent the standard error of 3 to 6 independent experiments.

We transformed [*psi^−^*] and [*PSI^+^*] derivatives of the A1–A8 strains with pSM1152, a plasmid that allows the expression of HA-tagged CFTR under the control of the constitutive *PGK1* promoter [68]. The degradation of CFTR through ERAD was then monitored by cycloheximide-chase as described in Material and Methods [67], [68] (Figure 11 and Supplemental Figure S2). CFTR was degraded at similar rates in the A2, A3, A5, A6, and A7 [*psi^−^*] strains and in a wild-type control (Figure 11 and Supplemental Figure S2). The only difference was observed for the A8 and to a lesser extent A1 and A4 strains where CFTR levels decreased faster upon cycloheximide treatment compared to the other strains (Figure 11). These results indicate that all the Ssa proteins are able to maintain CFTR in a soluble form that is competent for proteasomal degradation. However, subtle differences in the way these Ssa proteins interact with CFTR or with other ERAD components may account for the observed differences in CFTR degradation rates.

We then asked whether the degradation of CFTR could be affected by the presence of the [*PSI^+^*] prion and therefore repeated these experiments in the [*PSI^+^*] derivatives of the wild-type and A1–A8 strains expressing CFTR (except for A6 and A7 where [PSI^+^] propagation is too unstable). The expression of CFTR did not affect the prion phenotypes of our strains (data not shown). As shown in Figure 11 and Supplemental Figure S2, CFTR degradation in the wild-type strain and in the A2, A3, and A8 strains occurred at the same rate regardless of the psi-state. In the A5 and to a lesser extent A1 and A4 strains, the degradation of CFTR was slower in the [*PSI^+^*] derivatives. These data suggest that in those strains, the activity of Hsp70 is limiting to cope with both Sup35 aggregates and CFTR misfolding, or that the ubiquitin proteasome system may be challenged by the presence of cellular aggregates [69].

### Phenotypic differences among strains expressing individual Ssa proteins are not due to differences in Hsp104, Ydj1p or Sis1p abundance

We monitored the levels of Hsp70, Ydj1p, Sis1p and Hsp104 in [*psi^−^*] and [*PSI^+^*] derivatives of the strains expressing CFTR (Supplemental Figure S3), because differences in levels or activity of these chaperones affects different yeast prions and could account for our observed phenotypic differences. The levels of Ydj1p and Sis1p, two major regulators of Hsp70 in yeast, were similar in all the strains regardless of the presence of the prion (Supplemental Figure S3). The levels of Hsp104, while slightly higher in the A1–A8 strains compared to wild-type, where otherwise comparable and not affected by the prion (Supplemental Figure S3). The Ssa proteins were expressed from the same *SSA2* promoter and therefore Hsp70 levels should be similar in all the strains, although we can not rule out differences in protein turnover. The commercial anti-Hsp70 antibody we used (see Material and Methods) did not equally recognize each Ssa protein (Supplemental Figure S3) and it was therefore not possible to properly quantify Hsp70 levels in each strain. However, this antibody allowed us to show that Hsp70 levels were not affected by the presence of the prion (Supplemental Figure S3). Differences in Hsp70 levels among strains are unlikely to reflect the observed differences in growth rates and prion phenotypes because Ssa4p, Ssa5p and Ssa7p were detected at the same levels by our antibody, yet they conferred very different phenotypes. Similar protein levels were obtained in the same strains that do not contain the CFTR-expression plasmid (data not shown).

## Discussion

The question of a functional specialization among the cytosolic Hsp70 isoforms of the Ssa-subfamily has never been systematically tested, although several reports indicate such a possibility [11]. Here, we show that highly homologous intra- and inter-species Hsp70s have redundant yet clearly distinct functions. Not surprisingly, the four *S. cerevisiae* Ssa Hsp70s supported growth reasonably well, although the somewhat more divergent inducible Ssa3p and Ssa4p did not function as well as Ssa1p and Ssa2p. As anticipated, the interspecies orthologs Ssa5p-Ssa8p, whose divergence from Ssa1/2p is similar to that of Ssa3/4p, did not support growth of *S. cerevisiae* as well as Ssa1/2p. Somewhat unexpectedly, however, although Ssa5p-Ssa8p are significantly more homologous to each other, growth rates of strains A5–A8 ranged more widely. Here again the inducible isoforms (Ssa6 and Ssa7p) were less competent at supporting growth. The capacity of Ssa5p–Ssa8p to support growth of *Y. lipolytica* is unknown but our results suggest that they also differ with regard to essential cellular functions in their native context, and that Ssa6p has evolved more specialized function, perhaps related to differences in this organism's environment or metabolic activities.

Propagation of [*PSI^+^*] and [*URE3*] prions was affected differently by the different Hsp70s, which uncovered additional distinctions among the Hsp70s. For example, Ssa7p supported [*URE3*] well but [*PSI^+^*] very weakly. These differences could reflect differences in specific intrinsic Hsp70 activities, or in ways the Hsp70s interact with the prion proteins as substrates or with other components of the chaperone machinery. For example, the reduced curing of [*URE3*] by Ydj1p in strains A3 and A4 compared with A7 and A8 could be explained either by differences in affinity of these different Ssa proteins for Ure2p, or by differences in efficiency that Ydj1p interacts with the interspecies Ssa7p and Ssa8p than with Ssa3p and Ssa4p. These results also confirm our earlier conclusion that curing of [*URE3*] by Ydj1p requires a specific interaction with Hsp70 [63].

Changes in prion strength and mitotic stability reflect effects primarily on processes of growth and replication, respectively. The disparity between the wide variation in [*PSI^+^*] phenotype and the similarity in length of polymers, in particular with respect to strains A3 and A4, imply that the commonly used SDD-AGE assay cannot on its own distinguish effects on growth or fragmentation of prion polymers, and may therefore not be used as a predictive tool for prion phenotype. Similarly, we saw no correlation between [*PSI^+^*] phenotype and fluorescence pattern of our Sup35-GFP fusion protein, suggesting effects on prion phenotype were not related to specific changes in gross aggregation state of Sup35p. In fact, here again effects of Ssa3p and Ssa4p were indistinguishable despite their opposite effects on [*PSI^+^*]. Thus, although aggregation of Sup35-GFP into visible fluorescent aggregates is diagnostic of the presence of [*PSI^+^*], it was not useful for predicting prion phenotype, which raises caveats in the field regarding interpretation of such data with respect to how various factors influence propagation of prions. Together our data suggest that the visible aggregates are byproducts that may or may not be directly involved in prion propagation, a suggestion proposed previously [59], [60]. It is also possible that the different Hsp70s influence the ability of different types of Sup35p aggregates to participate in prion propagation.

Aside from the degree to which sequence specificity might determine differences in how chaperones interact with different prion proteins, the structural conformation of prion aggregates formed by them could contribute to determining which chaperone functions are more important for prion propagation of a particular prion. For example, if [*URE3*] prions are organized in a way that makes them less accessible to certain chaperones, then they might be more sensitive to changes in abundance or activity of these chaperones for certain aspects of their propagation. Differences in phenotype would then reflect differences in efficiency with which the different Hsp70s functionally interact, either positively or negatively, with other components of the disaggregation machinery.

The additional levels of distinction in Hsp70 activity uncovered by the prions did not necessarily correlate with Hsp70 function in essential cellular processes. For example, [*PSI^+^*] and [*URE3*] both propagated normally in A5 cells, which grew considerably slower than A3 cells, while in A3 cells [*PSI^+^*] was stronger than normal and [*URE3*] was weaker. Both of these Hsp70s functioned well with Hsp104 in refolding thermally denatured luciferase. We and others have found differences in Hsp104 functions with regard to prion propagation and thermotolerance [70]–[72], suggesting the disaggregation machinery interacts differently with amorphous aggregates than with highly ordered prion aggregates, and such differences might therefore be determined by specific Hsp70 components of the machinery. The prions therefore diagnose specific and non-essential Hsp70 activities that would be difficult to identify by growth phenotypes, and thus provide useful tools that will continue to help characterize the diverse interactions of the chaperone machinery in different cellular contexts.

Regardless of the mechanisms involved, the ability of the Hsp70s to support growth and prion propagation shows that the *Y. lipolytica* Ssa proteins functionally interact with other *S. cerevisiae* chaperones and co-chaperones to protect against protein aggregation, help proteins fold, facilitate protein transport, assist protein degradation and cooperate in protein disaggregation. It is not known if prions exist in *Y. lipolytica*, but our results imply that Ssa5p-Ssa8p are capable of functioning in a way that is compatible with supporting prion propagation in their native context. We anticipate our system also will be useful to assess various functions of combinations of interspecies chaperones and co-chaperones.

The degradation of CFTR through ERAD in yeast is compromised in strains that are defective for Hsp70 function [67], yet CFTR was degraded in all our strains, although with different efficiencies (Figure 11). This indicates that the intra- and inter-species Ssa proteins all interacted with CFTR and help maintain it in a soluble degradation-competent form [67], regardless of the growth rates of the corresponding strains. Importantly, our cycloheximide-chase experiments only allow us to monitor the degradation of a soluble pool of CFTR present at the ER-membrane. In yeast, CFTR was shown to be concentrated at defined sites in the ER membrane that were proposed to be Russell bodies, an ER sub-compartment in which misfolded proteins are stored and can be targeted for degradation [73]–[75]. Hence, we can not exclude that the different Hsp70 orthologs while having minor effects on the degradation of the soluble pool of CFTR, may differentially cause the aggregation of a fraction of CFTR and its targeting instead to aggresome-like bodies. While completely speculative, such an effect on CFTR aggregation could explain the slowed CFTR degradation rates observed in some (but not all) [*PSI^+^*] cells compared to the corresponding [*psi^−^*] cells. Indeed, in such a situation the ubiquitin proteasome system and the cellular quality control machineries may be overwhelmed or inhibited by both Sup35p and CFTR aggregates [69].

Our work clearly demonstrates that cytosolic Hsp70 orthologs have distinct functional properties despite a high degree of sequence homology. This functional specialization is probably needed to cope with the wide panel of substrates and functions that Hsp70 encounter in the cytosol and suggests an evolutionary pressure to maintain multiple Hsp70s in the eukaryotic cytosol, while most organelles (ER; mitochondria; chloroplast) generally have only one Hsp70 [11]. Deciphering the molecular basis of the functional specificities among Hsp70 orthologs has major implications in humans where at least six Hsp70 of the HSPA subfamily coexist [8], [11] that are implicated in conformational and cancer diseases. The distinctions we find, in particular with regard to differences in effects on growth and prion propagation, point to possible strategies for altering chaperone function to combat these diseases.

## Materials and Methods

### Strains media and plasmids

S. cerevisiae strains were G402 (MATa, kar1–1, SUQ5, ade2–1, his3Δ202, leu2Δ1, lys2, trp1Δ63, ura3–52 ssa1::KanMX, ssa2::HIS3, ssa3::TRP1, ssa4::ura3–1f/pRDW10 [44]), and related strain 1161 (MATα, kar1–1, SUQ5, P_DAL5_::ADE2, his3Δ202, leu2Δ1, trp1Δ63, ura3–52 ssa1::KanMX, ssa2::HIS3, ssa3::TRP1, ssa4::ura3–2f/pJ401 [38]). The ade2–1 strain 1135, which is isogenic to strain 1161, was used previously to characterize the S.cerevisiae Ssa proteins for ability to support growth and [PSI^+^] propagation [38]. The current study was initiated before strain 1135 was constructed and we used it only to confirm that any phenotypic differences of the Ssa proteins were not due to strain background (unpublished observations). Our wild type ade2–1 and P_DAL5_::ADE2 isogenic strains with intact SSA genes are 779–6A and 1075, respectively [38], [76]. The Y. lipolytica strain 136463 (MatB, scr1::ADE1, his-1, leu2, ura3 [77]) was used as the source of the SSA5–8 genes and to determine their expression patterns.

Plasmids pRDW10 (strain G402) [48] and pJ401 (strain 1161) [38] are *URA3*-based, single-copy plasmids with *SSA1* or *SSA2*, respectively, under control of their own promoters. The *LEU2*-based plasmids pC210 ( = pA1), pDCM62 ( = pA2), pA3 and pA4 are described [36], [38]. Plasmids pA5, pA6, pA7 and pA8 are pC210 with the coding region of *SSA5* (locus: YALI0F25289g; UniProt ID: Q6C0E9), *SSA6* (locus: YALI0E35046g; UniProt ID: Q6C3G5), *SSA7* (locus: YALI0D08184g; UniProt ID: Q6C9V0) and *SSA8* (locus: YALI0D22352g; UniProt ID: Q6C864) [11] on *Nde*I-*Sph*I fragments, in place of *SSA1* (oligonucleotides used for cloning are described in Supplemental Table S1). Plasmid p316GalYDJ, used for galactose-inducible overexpression of Ydj1, is a *URA3*-based single copy vector with *YDJ1* controlled by the Gal1-10 promoter. Plasmid p316CupNGMC is a *URA3*-based single-copy vector with the previously described *SUP35-GFP* fusion gene *NGMC* [60] under control of the *CUP1* promoter. Plasmid pDCM90 is a *URA3*-based single-copy plasmid containing a gene for expression of a thermolabile bacterial luciferase (LuxAB, [45]) on a *Cla*I-*Sma*I fragment.

Cultures were grown at 30°C unless indicated otherwise. Media were as described [38], [77]. Liquid YPAD contains excess adenine. Solid 1/2YPD medium has limiting adenine. Defined media contain 9 mg/l (limiting) or 400 mg/l (excess) adenine.

### Thermoresistance

Thermoresistance assays were done essentially as described in [40]. Briefly, 1 mL of yeast cells grown at 28°C to an OD_600__nm_∼0.15–0.3 were placed in a 52°C circulating water-bath. At the indicated time points, 100 µL aliquots were removed and transferred to a microfuge tube on ice. Serial dilutions for each sample were spread on YPAD plates and incubated at 28°C for 3–5 days. The viability at time zero was set to 100%.

### RT-PCR analysis

Total RNA was prepared from 50 mL cultures of a wild-type *Y. lipolytica* strain grown in the conditions indicated in the Results section and using the QIAGEN RNeasy kit. RNA preparations were devoid of genomic DNA contamination as standard PCR analysis failed to detect amplicons using the primers and conditions of the RT-PCR reactions described below (data not shown). Reverse transcription (RT) reactions were made using 1 to 100 ng of RNA, Ready-To-Go RT-PCR beads and were primed with an oligo-dT oligonucleotide according to the manufacturer’s instructions (GE Healthcare). The RT reaction was then split in two equal parts. Primers allowing the selective amplification of individual Hsp70 isoforms were added to one half of the reaction, and those allowing the amplification of the actin-encoding gene *Yl.ACT1* (accession no. Q9UVF3) were added to the second half of the reaction. PCR amplification was then performed for 35 cycles, using an annealing temperature of 52°C and according to the manufacturer’s instructions (GE healthcare). The primer pairs used for the RT-PCR analysis are described in Supplemental Table S1.

### Luciferase refolding

Luciferase refolding was assayed as described [45]. Log phase cells containing pDCM90 grown at 30°C in dextrose medium lacking uracil were first diluted to OD600 nm  = 0.2 into the same medium. Diluted cultures were incubated with shaking at 37°C for 30 minutes to induce expression of heat shock proteins, and then transferred to a 42°C water bath and incubated with shaking for one hour. Cycloheximide was added 50 minutes after shifting to 42°C to prevent synthesis of luciferase during the recovery period. Cultures were shifted to 25°C for 30 minutes and luciferase activity of 200 µl aliquots was measured in a Zylux luminometer (Model FB15) immediately after addition of 10 µl decanal (Sigma cat # D7384).

### Monitoring prions

We monitored [*PSI^+^*] in strain G402, which has the *ade2*–*1* nonsense mutation and thus requires exogenous adenine for growth. G402 cells are red when adenine is limiting due to accumulation of a metabolite of the adenine biosynthetic pathway. Depletion of the translation termination factor Sup35p into [*PSI^+^*] prion aggregates reduces efficiency of termination, which, in combination with the *SUQ5* tRNA, suppresses *ade2*–*1*. Thus, while G402 [*psi^−^*] cells that lack prions require adenine and are red, G402 [*PSI^+^*] cells grow without adenine and are white. We monitored [*URE3*] in strain 1161 using the *P_DAL5_::ADE2* reporter, in which the wild type *ADE2* gene is regulated by the *DAL5* promoter [78], [79]. The nitrogen catabolite regulator Ure2p represses the *DAL5* promoter when a good source of nitrogen is available, so on standard growth media containing ammonium *ADE2* expression is repressed and, like *ade2*–*1* mutants, cells require exogenous adenine to grow and are red when adenine is limiting. When [*URE3*] is present, depletion of Ure2p into prion aggregates relieves repression of the *DAL5* promoter so *ADE2* is expressed and cells grow without adenine and are white.

### Semi-denaturing detergent agarose gel electrophoresis (SDD-AGE)

Cells with p316CupNGMC were grown in dextrose medium lacking uracil and diluted to OD_600__nm_  = 0.05 into the same medium and into identical medium with 25 µM CuSO_4_. Cells grown for 15 hours were harvested for analysis. SDD-AGE was performed as described [60], [80]. Briefly, cell lysates were incubated with 2% SDS at 45°C for 10 min and then proteins were electrophoretically separated in 1.8% agarose gels containing 0.1% SDS. Proteins were then electrophoretically transferred to nylon membranes and probed with anti-GFP antibody.

### Fluorescence microscopy

Cells from the agarose gel experiments at the time of harvesting were used. Fluorescent images were captured using an Olympus BX61 microscope with IPlab software and were processed using Adobe Photoshop software.

### Curing of [URE3] by Ydj1p overexpression

Cells containing p316CupNGMC were grown in dextrose medium lacking uracil and diluted to OD_600__nm_  = 0.02 into the same medium and into similar medium containing 1% raffinose and 2% galactose in place of dextrose. Cultures were grown to OD_600__nm_  = 1.5–2 and then diluted and spread onto 1/2YPD to obtain 200–500 colonies per plate. Red colonies were scored as having lost [*URE3*]. After scoring, the plates were replica-plated onto –Ade and 1/2YPD plates containing 3 mM guanidine to verify that white colonies had the prion. Cultures of cells containing empty vector were treated identically as controls to monitor basal prion stability.

### Induction of [PSI^+^] by transformation of yeast spheroplasts using cell extracts

Cell extracts were made as described in [81]. Briefly, 50 mL of yeast cultures grown overnight at 30°C were centrifuged and the cell pellet suspended in 500 µL of STC buffer (1,2 M sorbitol, 10 mM CaCL_2_, 10 mM Tris. Cl, pH 7.5) containing a protease inhibitor cocktail (Roche Diagnostics). Cells were broken by repeated glass-beads beating on ice and the cellular debris removed by two centrifugation steps (5 min at 1100 g and at 4°C). Cell extracts were kept on ice and briefly sonicated (∼10 sec with a Branson Sonifier 150 and a microtip at 20% intensity) just before use. Transformation of yeast spheroplasts was performed as described in [58] with minor modifications. The indicated strains were grown to mid-log phase (OD_600__nm_ ∼0.6) in 50 mL of YPAD and spheroplasts were obtained as described [58] in 1 mL of STC buffer (1.2 M Sorbitol, 10 mM Tris-HCl pH 7.5, 10 mM CaCl_2_) and kept at 4°C until use (on the same day). Transformation mixtures contained 100 µl of spheroplasts, 100 µg/mL salmon sperm DNA, 20 µg/mL of the *URA3*-based pRS316 plasmid, and 300 µg/mL of the indicated yeast crude extract. Transformation mixtures without pRS316, without cell extract or containing extracts derived from a [*psi^−^*] strain were used as controls. After 30 min at room temperature, 9 volumes of PEG8000 buffer (10 mM Tris–HCl, pH 7.5, 20% PEG 8000, 10 mM CaCl_2_) were added and the mixture incubated for 30 min at room temperature. Spheroplasts were then collected by centrifugation (5 min at 400 g), suspended in 150 µL of SOS buffer (1M sorbitol, 7 mM CaCl2, 0.25% yeast extract, 0.5% bactopeptone, 0.5% glucose) and incubated for 30 min at 30°C. The cells were then gently mixed with 7 mL of top agar (synthetic complete medium containing 2.5% agar, 1M sorbitol and required aminoacids except adenine and uracil) kept at 45°C. The top-agar was poured onto defined medium plates lacking uracil and containing 6 mg/mL adenine and 1M sorbitol. The plates were then incubated at 30°C for up to ten days. [*PSI^+^*] candidate colonies were isolated from the transformation plates and further checked for prion propagation on defined medium lacking uracil and containing trace amounts of adenine or 1/2 YPD plates, and for prion curability on YPD plates containing 3 mM guanidium chloride.

### CFTR degradation assay and Western blot analysis

The degradation of HA-tagged CFTR was performed as described [67], [68] with minor modifications. Briefly, yeast cells carrying the pSM1152 plasmid were grown to exponential phase (OD_600__nm_ ∼0.4–0.6) at 28°C then cycloheximide was added at a final concentration of 200 µg/mL. At each indicated time point, an aliquot of 3 OD_600__nm_ of cells was taken and total protein extracts were prepared with the sodium hydroxide/trichloroacetic acid (NaOH/TCA) method as described [68]. Proteins were resuspended in 60 µl of TCA sample buffer (80 mM Tris-Cl, pH 8.0, 8 mM EDTA, 120 mM dithiothreitol, 3.5% SDS, 0.29% glycerol, 0.08% Tris base, 0.01% bromphenol blue), incubated at 37°C for 30 min and resolved by SDS-PAGE followed by blotting on a nitrocellulose membrane. Each membrane was then cut in half (approximately at the 90 kDa limit); the upper half was immunoblotted with anti-HA-peroxidase mouse monoclonal antibodies (Roche Applied Science) to detect CFTR, and the lower half with anti-BiP rabbit polyclonal antibodies as a loading control (a generous gift from Jeffrey L. Brodsky, University of Pittsburgh). Immunoblots were analyzed by PhosphorImager and quantified with the ImageQuant software (Molecular Dynamics).

To assess the levels of Hsp70, Ydj1p, Sis1p, Hsp104 and RPP0, extracts were similarly prepared, without adding cycloheximide to log phase cultures, and analyzed by western blot. The polyclonal rabbit anti-Ydj1p, anti-Sis1p, anti-Hsp104p and anti-RPP0 antibodies were a generous gift from Ronald Melki (CNRS, Gif-sur-Yvette). The monoclonal mouse anti-Hsp70/Hsc70 antibody was purchased from Stressgen Biotechnologies, Victoria, Canada (catalog number SPA-822).

## Supporting Information

Figure S1 Multiple Alignments of the *Saccharomyces cerevisiae* and *Yarrowia lipolytica* Ssa proteins. The alignments were generated using ClustalX 2.0.9 and GeneDoc V2.6.
(1.44 MB PDF)

Figure S2 CFTR degradation monitored by cycloheximide-chase. Representative gels for the quantifications shown in Figure 11 (see Material and Methods for details). Immunoblots against the endoplasmic reticulum protein BiP were used as loading controls.
(5.59 MB TIF)

Figure S3 Steady-state protein levels in strains expressing individual Ssaps. Total protein extracts were prepared from the indicated strains as described in figure 11 legend (without the addition of cycloheximide). Equal amounts of proteins were analyzed by SDS-PAGE and immunoblotting with antibodies against the indicated proteins.
(3.51 MB TIF)

Table S1 Oligonucleotides used in this study.
(0.04 MB DOC)
