# Supplementary figures and images for: Function of SSA Subfamily of Hsp70 Within and Across Species Varies Widely in Complementing Saccharomyces cerevisiae Cell Growth and Prion Propagation
Source: PLoS One. 2009 Aug 14;4(8):e6644. doi: 10.1371/journal.pone.0006644 (PMC2721632; doi:10.1371/journal.pone.0006644)

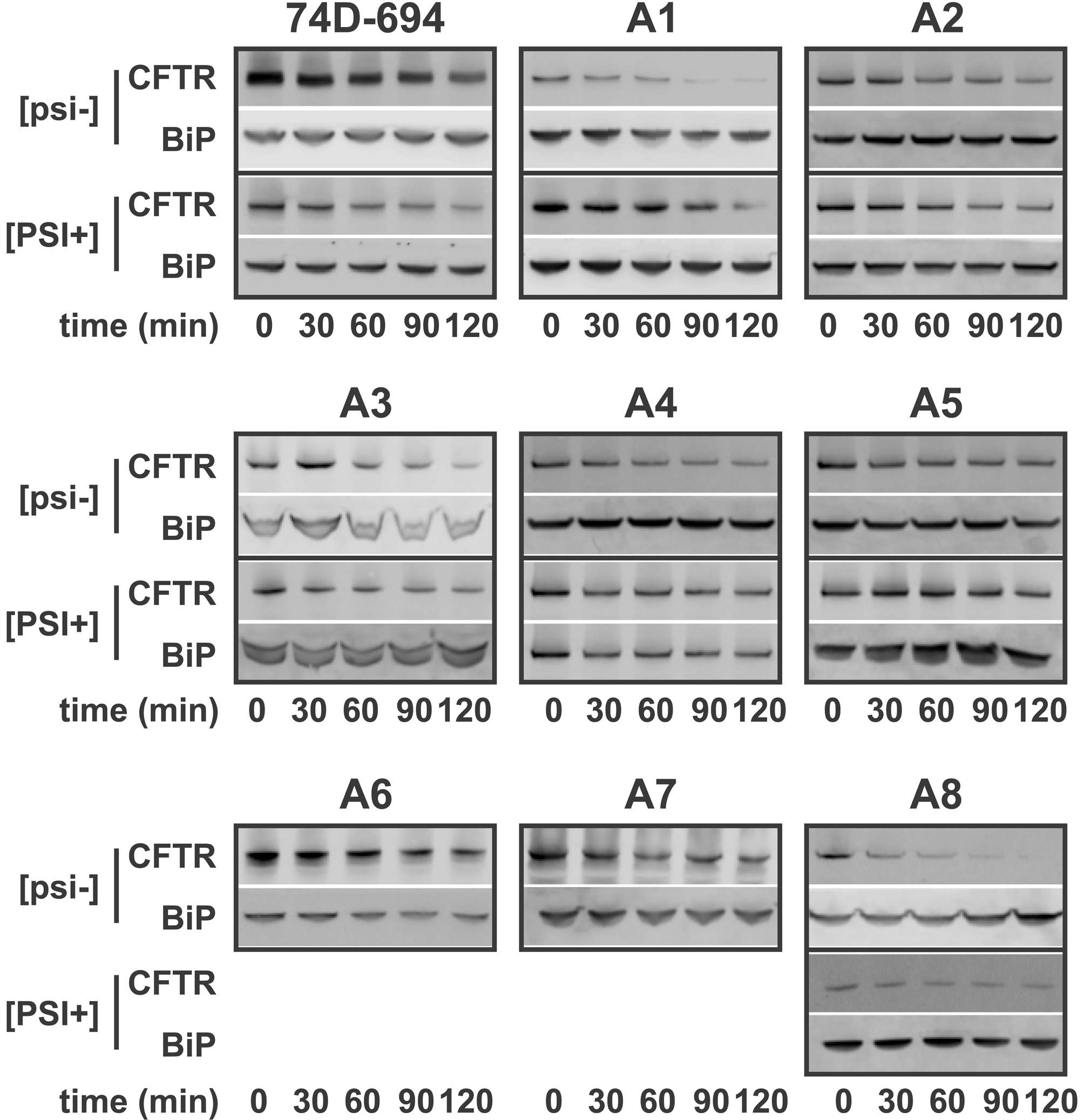

Supplement: Figure S2 — CFTR degradation monitored by cycloheximide-chase. Representative gels for the quantifications shown in Figure 11 (see Material and Methods for details). Immunoblots against the endoplasmic reticulum protein BiP were used as loading controls. (5.59 MB TIF) [file pone.0006644.s002.tif]

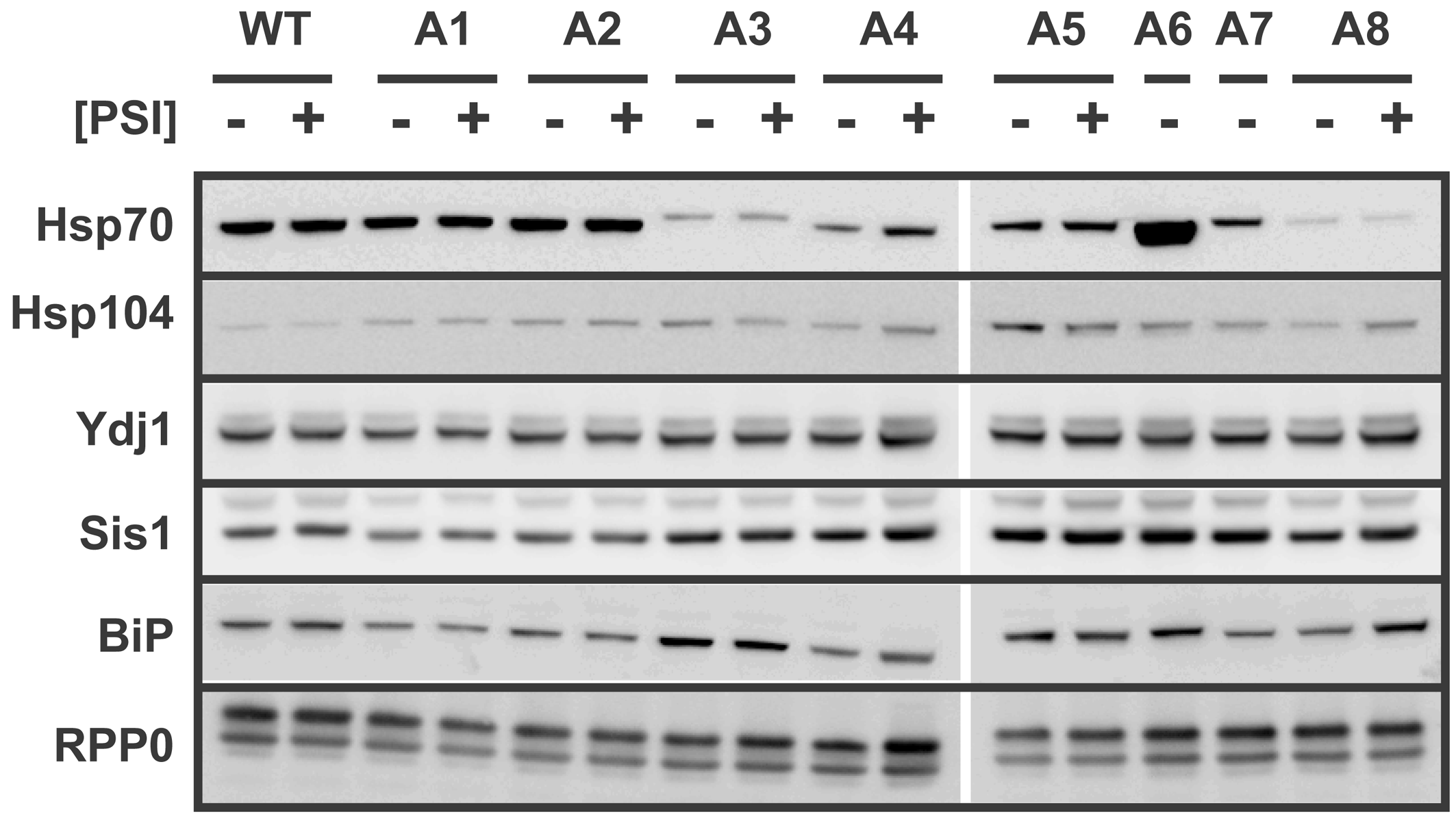

Supplement: Figure S3 — Steady-state protein levels in strains expressing individual Ssaps. Total protein extracts were prepared from the indicated strains as described in figure 11 legend (without the addition of cycloheximide). Equal amounts of proteins were analyzed by SDS-PAGE and immunoblotting with antibodies against the indicated proteins. (3.51 MB TIF) [file pone.0006644.s003.tif]
