# Supplementary material for: Function of SSA Subfamily of Hsp70 Within and Across Species Varies Widely in Complementing Saccharomyces cerevisiae Cell Growth and Prion Propagation
Source: PLoS One. 2009 Aug 14;4(8):e6644. doi: 10.1371/journal.pone.0006644 (PMC2721632; doi:10.1371/journal.pone.0006644)
Supplement: Table S1 — Oligonucleotides used in this study. (0.04 MB DOC) [file pone.0006644.s004.doc]

| name | oligonucleotide sequence | purpose |
| --- | --- | --- |
| SSA5-A ^a^ | GGGAATTCCATATGCCTAACACATCTGTAGGAATTGACCTG | cloning in pC210 |
| SSA5-B ^a^ | GAG**GCATGC**TTAGTCGACCTCCTCGACAGTGGGG | cloning in pC210 |
| SSA6-A ^a^ | GGGAATTCCATATGTCTAAAGCAGTAGGAATCGATCTTGG | cloning in pC210 |
| SSA6-B ^a^ | GAG**GCATGC**TTAGTCAACCTCCTCAACGGTGGG | cloning in pC210 |
| SSA7-A ^a^ | GGGAATTCCATATGTCCAAAGCAGTAGGAATTGATCTTGG | cloning in pC210 |
| SSA7-B ^a^ | GAG**GCATGC**TTAGTCAACCTCCTCAACGGTGGG | cloning in pC210 |
| SSA8-A ^a^ | GGGAATTCCATATGTCTAAAGCCGTCGGAATCGATCTTGG | cloning in pC210 |
| SSA8-B ^a^ | GAG**GCATGC**TTAGTCAACCTCCTCAACGGTGGG | cloning in pC210 |
| ACT1-5 | TCGTTATCGATAACGGATCC | RT-PCR |
| ACT1-R | TAGTAACACTTTCGGTGGAC | RT-PCR |
| SSA5-5 | TCGATCCTTACAACACATTTCAACAACAAAAATGCC | RT-PCR |
| SSA5-R | ACTAAAATACTTAGTCGACC | RT-PCR |
| SSA6-5 | CACAACACGAAAGAAACACATTACAACAACATGTC | RT-PCR |
| SSA6-R | ATACAAGGAATCCCTTAGTC | RT-PCR |
| SSA7-5 | AAGATTCTCTCCACAAAAACTAACTGAAACACAATGTCC | RT-PCR |
| SSA7-R | CATGCACAAGAAGATTAGTC | RT-PCR |
| SSA8-5 | GCTGGTCCTGGACTTTCTTCTTCTCCATACAACCACC | RT-PCR |
| SSA8-R | TACCTAGAAGTGATTTAGTC | RT-PCR |
| *Yl.*SSB1-5 | TAACAAAATGAGTGAAGGAACTTTTGCTGGAGC | RT-PCR |
| *Yl.*SSB1-R | ACTTAACTTATCGGGTAGCC | RT-PCR |

^a^ these primers contain an *Nde*I (underlined) or a *Sph*I (bold) site used for cloning
