# Supplementary material for: Function of SSA Subfamily of Hsp70 Within and Across Species Varies Widely in Complementing Saccharomyces cerevisiae Cell Growth and Prion Propagation
Source: PLoS One. 2009 Aug 14;4(8):e6644. doi: 10.1371/journal.pone.0006644 (PMC2721632; doi:10.1371/journal.pone.0006644)
Supplement: Figure S1 — Multiple Alignments of the Saccharomyces cerevisiae and Yarrowia lipolytica Ssa proteins. The alignments were generated using ClustalX 2.0.9 and GeneDoc V2.6. (1.44 MB PDF) [file pone.0006644.s001.pdf]

```
                   *        20         *        40         *        60         *        80         *       100
SSA1 : -MSKAVGIDLGTTYSCVAHFANDRVDIIANDQGNRTTPSFVAFTDTERLIGDAAKNQAAMNPSNTVFDAKRLIGRNFNDPEVQADMKHFPFKLIDVDGKP :  99
SSA2 : -MSKAVGIDLGTTYSCVAHFSNDRVDIIANDQGNRTTPSFVGFTDTERLIGDAAKNQAAMNPANTVFDAKRLIGRNFNDPEVQGDMKHFPFKLIDVDGKP :  99
SSA3 : -MSRAVGIDLGTTYSCVAHFSNDRVEIIANDQGNRTTPSYVAFTDTERLIGDAAKNQAAINPHNTVFDAKRLIGRKFDDPEVTTDAKHFPFKVISRDGKP :  99
SSA4 : -MSKAVGIDLGTTYSCVAHFANDRVEIIANDQGNRTTPSYVAFTDTERLIGDAAKNQAAMNPHNTVFDAKRLIGRKFDDPEVTNDAKHYPFKVIDKGGKP :  99
SSA5 : MPNTSVGIDLGTTYSCVAHFANDRVEIIANDQGNRTTPSFVAFTDTERLIGDAAKNQAAMNPSNTVFDAKRLIGRKFSDPEVQNDAKHFPFKIVDKGGKP : 100
SSA6 : -MSKAVGIDLGTTYSCVAHFANDRVEIIANDQGNRTTPSFVAFTDTERLIGDAAKNQAAMNPANTVFDAKRLIGRKFDDPEVQNDAKHFPFKIIDKAGKP :  99
SSA7 : -MSKAVGIDLGTTYSCVAHFANDRVEIIANDQGNRTTPSFVAFTDTERLIGDAAKNQAAMNPVNTVFDAKRLIGRKFTDEEVQTDAKHFPFKIVDKAGKP :  99
SSA8 : -MSKAVGIDLGTTYSCVAHFANDRVEIIANDQGNRTTPSFVAFTDTERLIGDAAKNQAAMNPANTVFDAKRLIGRKFSDPEVQNDAKHFPFKIVDKGGKP :  99

                   *       120         *       140         *       160         *       180         *       200
SSA1 : QIQVEFKGETKNFTPEQISSMVLGKMKETAESYLGAKVNDAVVTVPAYFNDSQRQATKDAGTIAGLNVLRIINEPTAAAIAYGLDKKGK-EEHVLIFDLG : 198
SSA2 : QIQVEFKGETKNFTPEQISSMVLGKMKETAESYLGAKVNDAVVTVPAYFNDSQRQATKDAGTIAGLNVLRIINEPTAAAIAYGLDKKGK-EEHVLIFDLG : 198
SSA3 : VVQVEYKGETKTFTPEEISSMVLSKMKETAENYLGTTVNDAVVTVPAYFNDSQRQATKDAGTIAGMNVLRIINEPTAAAIAYGLDKKGRAEHNVLIFDLG : 199
SSA4 : VVQVEYKGETKTFTPEEISSMILTKMKETAENFLGTEVKDAVVTVPAYFNDSQRQATKDAGTIAGLNVLRIINEPTAAAIAYGLDKKSQKEHNVLIFDLG : 199
SSA5 : NIEVEFKGETKVFTPEEISSMILTKMKETAEGYLGGKVTDAVITVPAYFNDSQRQATKDAGLIAGLNVQRIINEPTAAAIAYGLDKKEAGERNVLIFDLG : 200
SSA6 : NIEVEFKGETKVFTPEEISSMILTKMKETAEGYLGTKVNDAVITVPAYFNDSQRQATKDAGLIAGLNVQRIINEPTAAAIAYGLDKKETGERNVLIFDLG : 199
SSA7 : NIEVEFKGETKVFTPEEISSMILTKMKETAEGYLGGKVTDAVITVPAYFNDSQRQATKDAGLIAGLNVQRIINEPTAAAIAYGLDKKEEGERNVLIFDLG : 199
SSA8 : VIQVEFKGEQKTFTPEEISSMILVKMKEIAEAYLGGKVNDAVITVPAYFNDSQRQATKDAGLIAGINVLRIINEPTAAAIAYGLDKKGQGEQNVLIFDLG : 199

                   *       220         *       240         *       260         *       280         *       300
SSA1 : GGTFDVSLLFIEDGIFEVKATAGDTHLGGEDFDNRLVNHFIQEFKRKNKKDLSTNQRALRRLRTACERAKRTLSSSAQTSVEIDSLFEGIDFYTSITRAR : 298
SSA2 : GGTFDVSLLSIEDGIFEVKATAGDTHLGGEDFDNRLVNHFIQEFKRKNKKDLSTNQRALRRLRTACERAKRTLSSSAQTSVEIDSLFEGIDFYTSITRAR : 298
SSA3 : GGTFDVSLLSIDEGVFEVKATAGDTHLGGEDFDNRLVNHLATEFKRKTKKDISNNQRSLRRLRTAAERAKRALSSSSQTSIEIDSLFEGMDFYTSLTRAR : 299
SSA4 : GGTFDVSLLSIDEGVFEVKATAGDTHLGGEDFDSRLVNFLAEEFKRKNKKDLTTNQRSLRRLRTAAERAKRTLSSSAQTSIEIDSLFEGIDFYTSITRAR : 299
SSA5 : GGTFDVSLLSIEDGIFEVKATAGDTHLGGEDFDNRLVNHFVQEFKRKHKKDISTNQRALRRLRTACERAKRTLSSSAQTSIEIDSLYEGIDFYTSITRAR : 300
SSA6 : GGTFDVSLLSIEDGIFEVKATAGDTHLGGEDFDNRLVNHFVQEFKRKHKKDISTNQRALRRLRTACERAKRTLSSSAQTSIEIDSLYEGIDFYTSITRAR : 299
SSA7 : GGTFDVSLLSIEDGIFEVKATAGDTHLGGEDFDNRLVNHFVQEFKRKQKKDISTNQRALRRLRTACERAKRTLSSSAQTSIEIDSLYEGIDFYTSITRAR : 299
SSA8 : GGTFDVSLLSIEDGIFEVKATAGDTHLGGEDFDNRLVNHFVQEFKRKHKKDISTNQRALRRLRTACERAKRTLSSSAQTSIEIDSLYEGIDFYTSITRAR : 299

                   *       320         *       340         *       360         *       380         *       400
SSA1 : FEELCADLFRSTLDPVEKVLRDAKLDKSQVDEIVLVGGSTRIPKVQKLVTDYFNGKEPNRSINPDEAVAYGAAVQAAILTGDESSKTQDLLLLDVAPLSL : 398
SSA2 : FEELCADLFRSTLDPVEKVLRDAKLDKSQVDEIVLVGGSTRIPKVQKLVTDYFNGKEPNRSINPDEAVAYGAAVQAAILTGDESSKTQDLLLLDVAPLSL : 398
SSA3 : FEELCADLFRSTLEPVEKVLKDSKLDKSQIDEIVLVGGSTRIPKIQKLVSDFFNGKEPNRSINPDEAVAYGAAVQAAILTGDQSTKTQDLLLLDVAPLSL : 399
SSA4 : FEELCADLFRSTLEPVEKVLADSKLDKSQIDEIVLVGGSTRIPKVQKLVSDFFNGKEPNRSINPDEAVAYGAAVQAAILTGDQSSTTQDLLLLDVAPLSL : 399
SSA5 : FEELCQDLFRGTLEPVEKVLRDSKIDKSAVNEIVLVGGSTRIPKVQKLVSDFFNGKEPNRSINPDEAVAYGAAVQAAILSGDTSSSTQDLLLLDVAPLSL : 400
SSA6 : FEELCQDLFRGTLEPVEKVLKDAKMDKASVNEIVLVGGSTRIPKVQKLVSDFFNGKELNRSINPDEAVAYGAAVQAAILSGDTSSSTQDILLLDVAPLSL : 399
SSA7 : FEELCQDLFRGTLEPVEKVLRDAKMDKSSVNEIVLVGGSTRIPKVQKLVSDYFNGKEPNRSINPDEAVAYGAAVQAAILSGDTSSKTQDLLLLDVAPLSL : 399
SSA8 : FEELCQDLFRGTIEPVEKVLKDAKIDKASVNEIVLVGGSTRIPKIQKLVSDFFNGKEPNRSINPDEAVAYGAAVQAAILTGDTSSKTQDLLLLDVAPLSL : 399

                   *       420         *       440         *       460         *       480         *       500
SSA1 : GIETAGGVMTKLIPRNSTISTKKFEIFSTYADNQPGVLIQVFEGERAKTKDNNLLGKFELSGIPPAPRGVPQIEVTFDVDSNGILNVSAVEKGTGKSNKI : 498
SSA2 : GIETAGGVMTKLIPRNSTIPTKKSEVFSTYADNQPGVLIQVFEGERAKTKDNNLLGKFELSGIPPAPRGVPQIEVTFDVDSNGILNVSAVEKGTGKSNKI : 498
SSA3 : GIETAGGIMTKLIPRNSTIPTKKSETFSTYADNQPGVLIQVFEGERTRTKDNNLLGKFELSGIPPAPRGVPQIDVTFDIDANGILNVSALEKGTGKSNKI : 499
SSA4 : GIETAGGIMTKLIPRNSTIPTKKSEVFSTYADNQPGVLIQVFEGERTRTKDNNLLGKFELSGIPPAPRGVPQIEVTFDIDANGILNVSAVEKGTGKSNKI : 499
SSA5 : GIETAGGVMTKLIPRNSTIPTKKSETFSTYADNQPGVLIQVFEGERAQTKDNNILGKFELSGIPPAPRGVPQIEVTFDVDANGILNVSAVEKGTGKTQQI : 500
SSA6 : GIETAGGVMTKLIPRNSTIPTKKSETFSTYADNQPGVLIQVFEGERAQTKDNNILGKFELSGIPPAPRGVPQIEVTFDVDANGILNVSAVEKGTGKTQQI : 499
SSA7 : GIETAGGVMTKLIPRNSTIPTKKSETFSTYADNQPGVLIQVFEGERAQTKDNNILGKFELTGIPPAPRGVPQIEVTFDVDANGILNVSAVEKGTGKTQQI : 499
SSA8 : GIETAGGVMTKLIPRNSTIPTKKSETFSTYADNQPGVLIQVFEGERAQTKDNNILGKFELSGIPPAPRGVPQIEVTFDLDANGILSVSAVEKGTGKTQQI : 499

                   *       520         *       540         *       560         *       580         *       600
SSA1 : TITNDKGRLSKEDIEKMVAEAEKFKEEDEKESQRIASKNQLESIAYSLKNTISEAG--DKLEQADKDTVTKKAEETISWLDSNTTASKEEFDDKLKELQD : 596
SSA2 : TITNDKGRLSKEDIEKMVAEAEKFKEEDEKESQRIASKNQLESIAYSLKNTISEAG--DKLEQADKDAVTKKAEETIAWLDSNTTATKEEFDDQLKELQE : 596
SSA3 : TITNDKGRLSKDDIDRMVSEAEKYRADDEREAERVQAKNQLESYAFTLKNTINEASFKEKVGEDDAKRLETASQETIDWLDASQAASTDEYKDRQKELEG : 599
SSA4 : TITNDKGRLSKEDIDKMVAEAEKFKAEDEQEAQRVQAKNQLESYAFTLKNSVSENNFKEKVGEEDARKLEAAAQDAINWLDASQAASTEEYKERQKELEG : 599
SSA5 : TITNDKGRLSKEDIERMVNDAEKYKEQDEAEAARIAAKNGLESYAYSLKNTLSEEKFKEKVDEADREKLEKAINETIEFLDSTQSGATEEYSDKQKELEG : 600
SSA6 : TITNDKGRLSKEEIERMVNDAEKYKDEDEKEAARIAAKNGLESYTYSLKNTFSEEKFKEKVDEAEREKLEKAINETIEFLDATQSGATEEYSDKQKELEG : 599
SSA7 : TITNDKGRLSKEDIERMVSEAEKYKDEDEKEAARIAAKNGLESYAYSLKNTLSEEKFKEKVDEADREKLEKAINETIEFLDSTQSGATEEYSDKQKELEG : 599
SSA8 : TITNDKGRLSKEEIDRMVNDAEKYKEQDEAEAKRIAAKNGLESYAYSLKNTLSEEKFKEKVEEPEREKLEKAINETIEFLDATQSGTTEEYADKQKELEG : 599

                   *       620         *       640         *
SSA1 : IANPIMSKLYQA--GGAPGGAAG-GAPGGFPG---GAPPAP--EAEGPTVEEVD  : 642
SSA2 : VANPIMSKLYQA--GGAPEGAA----PGGFPG---GAPPAP--EAEGPTVEEVD  : 639
SSA3 : IANPIMTKFYGAGAGAGPGAGES----GGFPGSMPNSGATGGEDTGPTVEEVD  : 649
SSA4 : VANPIMSKFYGAAGGAPGAGP--------VPG--AGAGPTG-APDNGPTVEEVD- : 642
SSA5 : VANPILMKFYGA-EGGAPGGMPGAGGPGGFPGAGAGGAPPPGAGGEGPTVEEVD  : 653
SSA6 : IANPILMKFYGA-EGGAPGGMPG----GGMPGS--GAAPG---GGEGPTVEEVD  : 643
SSA7 : VANPILMKFYGA-EGGAPGGMPGGA-PGGFPGAGEGAAPG---GGEGPTVEEVD  : 648
SSA8 : VANPILMKFYGA-EGGAPGGAP--GGPGGFPGAGAGG-PPPGAGGDGPTVEEVD  : 649
```
